# Nalmefene and naltrexone reduce alcohol intake via selective efficacy in subpopulations distinguished by behavioral and blood-based biomarkers

**DOI:** 10.1038/s43856-025-01369-6

**Published:** 2026-01-14

**Authors:** Zahra Z. Farahbakhsh, Alex R. Brown, Suzanne O. Nolan, Snigdha Mukerjee, Cody A. Siciliano

**Affiliations:** Vanderbilt University, Department of Pharmacology, Vanderbilt Brain Institute, Vanderbilt Center for Addiction Research, Nashville, TN USA

**Keywords:** Addiction, Motivation

## Abstract

**Background:**

The relative efficacies of nalmefene versus naltrexone for alcohol use disorder is the subject of intense and ongoing debate. The two pan-opioid receptor ligands differ primarily in actions at the kappa opioid receptor, where naltrexone acts as an antagonist and nalmefene acts as a partial agonist. Parallel clinical trials for nalmefene or naltrexone have produced widely disparate outcomes and a marked lack of consensus regarding which of the compounds should be used for the treatment of alcohol use disorder.

**Methods:**

Here we leveraged a mouse model (n = 56 male C57BL/6 J) to directly compare the efficacy of nalmefene and naltrexone within-subject. After acquiring operant responding for ethanol, each subject underwent four treatment block conditions: nalmefene (0.1 mg/kg i.p.), naltrexone (1.0 mg/kg i.p.), the selective kappa opioid receptor agonist U50,488 (1.0 mg/kg i.p.) and placebo (saline 10 ml/kg i.p.). Each treatment block consisted of an ethanol self-administration session followed by two subsequent sessions of punished (quinine adulterated) ethanol self-administration sessions with treatment given 30 min prior to each session.

**Results:**

We show that nalmefene and naltrexone have similar efficacy in reducing ethanol consumption, whereas U50,488 increases ethanol consumption. Despite similar effects in aggregate analyses, nalmefene- and naltrexone-induced reductions in drinking are driven by fully separate subpopulations which do not show any beneficial response to the non-preferred compound and display markedly different behavioral phenotypes prior to treatment. A predictive model based on circulating biogenic amines allows for high accuracy classification of nalmefene- versus naltrexone-responders.

**Conclusion:**

Together, these results provide a roadmap for improving alcohol use disorder treatment outcomes via precision application of existing compounds.

## Plain Language Summary

Nalmefene and naltrexone are two drug treatments for alcohol use disorder, but disagreement exists on which is more effective. Despite ongoing controversy, treatment outcomes have only been compared across separate studies, rather than head-to-head. Using a preclinical model where mice learn to press a lever for alcohol, we treated each mouse with nalmefene, naltrexone, and placebo control. While both drugs equally reduced alcohol consumption on average, individual-level analysis revealed two distinct subpopulations: one responding to nalmefene but not naltrexone, and vice versa. Using machine learning on a single blood sample, we could predict which treatment would be more effective for each individual. This work suggests treatment outcomes for alcohol use disorder could be improved by using predictive models to deliver the right treatment to the right person at the right time.

## Abstract (web summary)

Farahbakhsh et al. directly compare nalmefene and naltrexone efficacy within subjects using a mouse model of alcohol self-administration. Both drugs reduce drinking equally overall but have efficacy in distinct subpopulations, and which treatment will be effective can be predicted based on behavioral phenotype and circulating biomarkers.

## Introduction

Alcohol use disorder (AUD) is a highly heterogenous disorder across its development, presentation, and treatment^1^. Not all individuals who consume alcohol go on to meet criteria for an AUD, but even among those that do, there is a spectrum of symptomatologies, as the presence of any combination of 2 or more of 11 symptoms qualifies a diagnosis for the disorder^2^. Response to treatment interventions is also highly variable and relapse rates in treated individuals are estimated to fall between 30 and 90%, with a wide range of relapse severity and timecourse^3–6^. This heterogeneity has prompted a push for a precision medicine approach, where the likelihood of treatment response for an individual is predicted based on personal characteristics that can be quantified in the clinic (e.g., genotype, circulating biomarkers, symptom presentation)^1,7–11^. Two clinically used drugs with similar mechanisms of action, naltrexone and nalmefene, have some efficacy for reducing drinking when prescribed without a personalized strategy^12,13^. Unlike naltrexone, nalmefene is not Food and Drug Administration (FDA)-approved for the treatment of AUD, despite its approval for AUD treatment by the European Medicines Agency since 2013 and the National Institute for Health and Care Excellence since 2014^14,15^. The comparative efficacy of these two compounds is the subject of intense and ongoing debate. Indeed, there are multiple reports claiming greater efficacy of nalmefene, some claiming equal efficacy, as well as some claiming that nalmefene has no efficacy in reducing drinking at all, and the approval or lack thereof by regulatory agencies have been met with staunch opposition^16–21^. Of note, though there has been limited direct comparison in human laboratory testing^22,23^, the two compounds have not been directly compared within a clinical trial^24^, and whether differentially responsive patient subpopulations might be driving the disparate findings across studies has not been directly tested.

Though naltrexone and nalmefene share similar chemical structures and both target the opioid receptor systems, the two compounds differ in receptor affinity, pharmacodynamic action, and clinical guidelines for prescription use. Naltrexone is widely regarded as a neutral pan-opioid antagonist (i.e., 0% intrinsic efficacy), though under some conditions inverse or partial agonism has been observed at mu receptors with reports ranging from approximately −10% to 40% intrinsic efficacy^25–29^. Nalmefene is also considered a neutral antagonist at mu and delta opioid receptors, but has higher affinity for kappa opioid receptors, both compared to naltrexone and relative to its affinity for mu and delta opioid receptors. In contrast to naltrexone, nalmefene is a partial agonist at kappa opioid receptors with ~25% intrinsic efficacy, though exact values can again vary by preparation^26,29–31^. Recommendations for prescription use also vary: naltrexone is taken daily with the goal of maintaining abstinence, whereas nalmefene treatment aims to reduce the amount of alcohol consumed – thus naltrexone is a daily medication, whereas nalmefene is taken “as-needed” on days when alcohol is expected to be consumed or when craving is high^32,33^.

Preclinical models provide a unique avenue for head-to-head comparisons of nalmefene and naltrexone, which can inform whether nalmefene should be further considered for AUD treatment in the United States and how both compounds can be best applied in the clinic. However, minimal preclinical work focuses on precision treatment strategies, and it remains unknown whether stable relationships between biobehavioral processes and pharmacotherapeutic responsivity can be reliably observed in rodent models of AUD, despite long-standing calls for research in this area from the alcohol field and the National Institutes of Health^34–38^. Additionally, nalmefene was recently FDA-approved for a new route of administration for opioid overdose treatment^39^ and could be readily repurposed for AUD^35^. In the European Union, the current recommendation for second-line intervention if patients are unresponsive to naltrexone is disulfiram, which can cause toxicity and has poor adherence^40^. In sum, if nalmefene and naltrexone have efficacy in different, identifiable subpopulations, substantial gains in overall treatment effectiveness could be possible in the short term.

To address this gap, we conducted within-subject comparison of nalmefene and naltrexone using an ethanol self-administration design in mice to directly compare the effect of these interventions. Longitudinal, within-subject testing revealed that the opioidergic treatment compounds nalmefene and naltrexone have comparable efficacy in reducing drinking at the population level. However, efficacy was attributable to entirely separate subpopulations: among nalmefene-responders, there was no effect of naltrexone and vice versa. Grouping individuals based on the most effective treatment compound, we found that these two subpopulations were also behaviorally distinct prior to treatment: nalmefene-responders consumed more ethanol, responded more for ethanol and for ethanol-conditioned stimuli, and were more likely to display a compulsive drinking phenotype (i.e., punishment-resistant drinking). Finally, a screen of circulating biogenic amines and amino acids allowed for the prediction of the more effective compound with 80% accuracy. Together, these results may account for the widely disparate clinical data regarding nalmefene vs naltrexone efficacies and suggest that precision application of these interventions could be achieved via blood-based screening.

## Methods

### Animals

Male C57BL/6J mice (strain #000664) were used for all experiments (Jackson Laboratory, Bar Harbor, ME). Animals were housed in groups of five in a temperature- and humidity-controlled animal facility on a 12-h reverse light-dark cycle (8 AM lights off, 8 PM lights on) with *ad libitum* access to water (all experiments were performed during the active phase/dark cycle). Animals arrived at 8 weeks of age and were allowed to acclimate to the facility for at least one week before any testing was performed, and were given *ad libitum* access to chow during this period. Following acclimation and throughout all experimental procedures, chow (Picolab 5L0D, LabDiet) was given daily at slightly above caloric requirements such that a healthy adult weight was maintained throughout the course of experiments (2.9–3 g/animal/day, corresponding to roughly 8.7 kcal^ME^/day)^41,42^. All experiments involving the use of animals were in accordance with NIH guidelines and approved by the Vanderbilt Institutional Animal Care and Use Committee.

The pharmacodynamics of and mechanisms of action of many kappa opioid receptor ligands are highly debated for female subjects^43–48^. Since we hypothesized that individual differences in efficacy between the compounds would be mediated by kappa opioid receptor differences and aimed to probe the system with a kappa opioid receptor agonist, we only used male mice in this study.

Sample size was determined to ensure accurate phenotyping of all animals, as such, a power analysis to a priori determine group sizes was not conducted, as each animal’s behavior dictated their stratification. Based on previous work, we used statistical modeling to determine that the minimum number of animals needed in a cohort to achieve stable phenotyping to be >12 animals (see ref. ^49^ for details). Given that in this study we had four treatment conditions (saline, naltrexone, nalmefene, U50,488), we used 48 mice as an estimate for total subject numbers needed. Expecting a roughly 10% subject attrition, based on task acquisition, we chose to run 60 mice across two cohorts of 30. Four mice were excluded for failing to meet the acquisition criteria outlined below.

### Ethanol self-administration: structured tracking of alcohol reinforcement (STAR)

All conditioning experiments were performed in an operant conditioning chamber (Skinner box, Med Associates) as previously described^49^. Chambers were equipped with ultrasensitive levers (Med Associates, ENV-312-3W) with wall lights above each port. A retractable sipper tube (Med Associates, ENV-352AW) was used to measure consumption and licks were detected by a resistance lickometer (Med Associates, ENV-250C). All sessions were run in the dark during the animals’ dark cycle, and mice were continuously monitored via overhead infrared cameras (Security Camera Warehouse).

Subjects were tested daily in one-hour sessions. 15% ethanol (v/v), prepared from 95% stock and diluted in ultrapure water, was used throughout all sessions. Mice were weighed at the conclusion of each daily session and fed after all animals completed their experiment for the day. Wall lights were illuminated at the start of each session (except for Magazine Training) and turned off during sipper extension. Responding on the inactive lever had no programmed consequence throughout (active side counter-balanced across animals).

### Operant acquisition

60 mice were run across two cohorts with minor experimental differences – any changes between the cohorts will be explicitly stated and all other conditions can be assumed to remain the same. During acquisition, animals were trained to respond to ethanol under increasing fixed-ratio schedules and shorter access periods. All mice ran through the same steps of acquisition in three phases, described below. If a mouse did not meet the criteria of a phase for three consecutive days, they were move back to the previous acquisition phase until the criteria were met again. If a mouse moved back a phase three times, they were considered a “non-learner” and dropped from the experiment (*n* = 4 non-learners excluded from analysis). See Supplementary Figs. 1, 2 for additional details and information regarding the paradigms and acquisition rates for the two cohorts of animals.

### Magazine training

Animals were placed in the operant conditioning chamber with the sipper tube extended throughout the session. Animals that reached ≥100 licks were moved on to operant conditioning the following day, while those that did not repeated magazine training on the subsequent session.

### Operant conditioning

Criteria 1: Responding on the active lever was reinforced by extension of the sipper for 30 s under a fixed-ratio 1 schedule. Animals were moved onto the second criteria once they reached ≥100 licks for two consecutive days.

Criteria 2: Responding continued to be reinforced under a fixed-ratio 1 schedule, but the access period was shortened to 10 s. Animals were moved onto operant discrimination once they reached ≥100 licks for two consecutive days.

### Operant discrimination

Response requirement was raised to a fixed-ratio 5 schedule for 10 s access. Animals were considered to have acquired once they displayed ≥70% responding rate on the “Active” side (Active/[Active+Inactive responses]) and reached ≥100 licks for two consecutive days. If a subject was returned to an earlier stage twice due to this acquisition criteria, they were allowed to progress on the third attempt as long as the 100 lick criteria was met.

### Cohort differences

Acquisition: A subset of animals (*n* = 26) underwent the same acquisition phases as outlined above with the following differences: sessions were capped at 100 licks or one hour, whichever came first (animals still had to reach 100 licks to meet daily criteria, but sessions ended immediately after the 100th lick, if reached). Operanda were illuminated nosepoke ports (Med Associates, ENV-313W) rather than levers. Lastly, after completion of acquisition, animals performed two additional phases prior to STAR Phenotyping to test the effect of binge drinking on phenotype development. First, they conducted 7 pre-binge sessions following the exact same format as the STAR Phenotyping sessions. Following the completion of the pre-binge epoch, animals were given two weeks of access to 15% ethanol in a two-bottle choice procedure as previously described^49^. Immediately after completion of the binge phase, animals performed the STAR Phenotyping sessions as described below.

### STAR phenotyping methodology

On the day following completion of acquisition, all animals ran once daily for one hour and sessions progressed in a fixed order regardless of performance. Throughout all sessions, responding was reinforced under a fixed-ratio 10 schedule by presentation of the sipper for 10 s. For days 1–3, the sipper contained 15% ethanol (v/v). For days 4–7, ethanol was adulterated with quinine at increasing concentrations for each session (250, 500, 750, and 1000 µM in 15% v/v ethanol).

To perform STAR phenotyping, two values are calculated for each animal and expressed as a percent of the sample mean: (1) subject’s average g/kg consumed over 3 ethanol only sessions [ethanol intake = (subject mean session 1–3 (g/kg)/mean of all subjects day 1–3 (g/kg))*100], and (2) subject’s average g/kg over 4 ethanol+quinine sessions [ethanol+quinine intake = (subject mean session 4–7 (g/kg)/ mean of all subjects day 4–7 (g/kg))*100]. Based on the calculated values for ethanol intake and ethanol+quinine intake animals are assigned to one of three phenotypes. Animals with below average values for both ethanol and ethanol+quinine are deemed “Low Drinkers” [ethanol <100% & ethanol + quinine <100%]; those with values above the average ethanol intake but below average ethanol + quinine were deemed “High Drinkers” [ethanol >100% & ethanol+quinine <100%]; animals with above average ethanol + quinine values were deemed ‘Compulsive Drinkers’ [ethanol + quinine >100%]. For visualization, the values for all animals were plotted as a scatter plot where x = [ethanol + quinine values] and y = [ethanol only values].

### Conditioned reinforcement

After pharmacological treatment, a subset of subjects was also tested under conditioned reinforcement parameters. During conditioned reinforcement, sipper tubes were left empty/dry. The first response on the active side on day 1 resulted in presentation of the dry sipper for 10 s. After the first presentation, the response requirement for 10 s access to the dry sipper was raised to fixed-ratio 10 for the remainder of the sessions. After conditioned reinforcement sessions, mice returned to the operant box for a single ethanol reintroduction session during which 15% ethanol was in the sipper.

### Pharmacological treatment

After all mice (with the exception of non-learners) completed ethanol self-administration training and baseline measurements of responding, they went through a series of treatment tests to observe the effects of naltrexone (1 mg/kg), nalmefene (0.1 mg/kg), and U50,488 (1 mg/kg) on unpunished and punished ethanol self-administration. The doses selected for nalmefene and naltrexone were based on work from Walker et al., demonstrating that 0.1 mg/kg and 1 mg/kg, respectively, were the most effective doses for reducing ethanol intake^50^. These doses have also been demonstrated to effectively antagonize both the mu and kappa opioid receptors via in vivo assays of opioid receptor activity. For example, 0.1 mg/kg nalmefene and 1 mg/kg naltrexone i.p. have been shown to attenuate morphine-induced locomotion and are sufficient to precipitate withdrawal after chronic morphine exposure^51,52^ suggesting effective antagonism of the mu opioid receptor at these doses. Further, 1 mg/kg naltrexone has been shown to block U50,488-induced analgesic effects suggesting that this dose is sufficient to also cause blockade of kappa opioid receptors^53^. Similarly, 0.1 mg/kg nalmefene subcutaneously is sufficient to antagonize the effects of the selective KOR agonist salvinorin A, demonstrating binding to kappa opioid receptors at this dose^54^. The dose of U50,488 chosen was based on prior work from Rose et al., which reported dose-dependent increases in ethanol intake during two-bottle choice drinking in mice with the greatest increase observed at 1–3 mg/kg^55^.

Separate experimenters prepared/injected the drugs in one setting, while a separate blinded experimenter performed behavioral testing and recorded daily data for subsequent plotting. Mice were intraperitoneally injected 30 min prior to each behavioral session and ran for one-hour sessions for three consecutive days under a fixed-ratio 10 schedule reinforced by 10 s of sipper access. On day 1 the sipper contained 15% ethanol and days 2 and 3 the ethanol was adulterated with 250 or 500 µM quinine, respectively. The quinine doses were selected as they are above and below the typical IC_50_ for quinine’s effects on alcohol consumption and therefore well-represent the linear portion of the dose response curve^49,56^. Between treatments, which were a minimum of 4 days apart to allow for drug washout, animals were in abstinence or ran in daily hour-long sessions until their consumption stabilized. All animals within a homecage were treated with the same solution, but treatments were counterbalanced across cages. One cohort (*n* = 26) underwent 3 separate saline control blocks and values from the 3 blocks were averaged to serve as the saline baseline values for those subjects. One cohort (*n* = 26) received treatment in the order of: naltrexone, nalmefene, and U50,488; the other cohort (*n* = 30) had treatment order counterbalanced.

Treatment response: Treatment response was calculated as a within-subject change from saline control sessions. Subjects were assigned to the nalmefene- or naltrexone-responder groups based on which compound induced the greatest change in consumption versus saline. If consumption was no different between the two compounds, subjects were assigned based on which compound induced the greatest change in licks versus saline, and in the case that there was no difference in licks, the change in active responses was compared.

### Drugs

Naltrexone hydrochloride (1 mg/kg, Tocris CAS#: 16676-29-2), nalmefene hydrochloride (0.1 mg/kg, Tocris CAS#: 58895-64-0), and U50,488H (1 mg/kg, NIDA Drug Supply Program) were dissolved in sterile saline (0.9% NaCl) and injected at a volume of 10 mL/kg intraperitoneally.

### Plasma collection

At least 5 months after receiving all pharmacological treatments, mice underwent three additional ethanol-only self-administration sessions. 24 h later, animals were anesthetized with isoflurane, rapidly decapitated, and trunk blood collected in potassium ethylenediaminetetraacetic acid-coated tubes. Blood was then centrifuged at 4 degrees at 3000 RPM (735 relative centrifugal force) for 10 min and plasma separated and aliquoted for liquid chromatography-mass spectrometry (LC-MS) analysis.

### Liquid chromatography-mass spectrometry

LC-MS Analysis was conducted to determine plasma levels of dopamine (DA), homovanillic acid (HVA), 5-hydroxyindoleacetic acid (5HIAA), serotonin (5HT), norepinephrine (NE), epinephrine (EPI), gamma-aminobutyric acid (GABA), glutamic acid (GLU), 3-methoxytyramine (3MT), acetylcholine, choline, octopamine, arginine (Arg), taurine, lysine (Lys), ornithine (Orn), proline (Pro), tryptophan (Trp), phenethylamine (PhEt), kynurenic acid (Kyn), cysteine (Cys), homocysteine, aspartic acid, alanine, tyrosine, butyric acid, and glutamine (Gln) (Supplementary Data 1). The detection limits were 50 pg/mL–1 µg/mL for each analyte. The goal of the screen was to identify predictive biomarkers, as opposed to mechanistic explanations, thus this panel was selected based on maximizing the number of analytes that could be detected in a single, low-volume sample using standard equipment and protocols, as well as capturing some of the more implicated analytes in the literature (i.e., monoamines and related metabolites).

#### Internal standard synthesis

Stock solutions of biogenic amines and amino acids (5 ng/µL each) were made in DI water and stored at −80 °C. To prepare internal standards, stock solutions were derivatized in a similar manner to samples using isotopically labeled benzoyl chloride. 50 µL of the amino acid stock solution was diluted with 200 µL acetonitrile. 100 µL each of 500 mM NaCO3 (aq) and 2% 13C6-BZC in acetonitrile was added to the solution. After two minutes, the reaction was stopped by the addition of 200 µL 20% acetonitrile in water containing 3% sulfuric acid and 400 µL water. This solution was stored in 10 µL aliquot at −80 °C. One aliquot was diluted 100× with 20% acetonitrile in water containing 3% sulfuric acid to make the working internal standard solution used in the sample analysis.

#### Benzoyl chloride derivatization and LC-MS analysis

Analytes in plasma were quantified using LC-MS methodology following derivatization with benzoyl chloride (BZC). 10 µL of plasma is diluted with 30 µL acetonitrile:water (80:20) and vortexed. The solution was spun at 3.5 g for 5 min to pellet proteins. 5 µL of supernatant was added 10 µL each of 500 mM NaCO_3_ (aq) and 2% BZC in acetonitrile. After two minutes, the reaction was stopped by the addition of 10 µL internal standard solution. The samples were then ready for LC-MS analysis. LC was performed on a 2.0 ×50 mm, 1.7 µm particle Acquity BEH C18 column (Waters Corporation, Milford, MA, USA) using a Waters Acquity UPLC. Mobile phase A was 15% aqueous formic acid and mobile phase B was acetonitrile. Samples were separated by a gradient of 98–5% of mobile phase A over 11 min at a flow rate of 600 µL/min prior to delivery to a SCIEX 6500+ QTrap mass spectrometer.

### Biomarker classification

27 analytes were quantified simultaneously from each sample using LC-MS. Small molecule neurotransmitters were measured in the Vanderbilt University Neurochemistry Core (Director, Dr. Ginger Milne). Circulating biomarker analysis was conducted in MATLAB using custom scripts, which iteratively trained and tested the model as depicted in Fig. 5. The classification tree was implemented using MATLAB’s fitctree function (https://www.mathworks.com/help/stats/fitctree.html) with Gini’s diversity index as the split criterion, a maximum of 100 splits, and automatic hyperparameter optimization enabled. The model was trained and tested across 500 independent iterations. On each iteration, 20% of the sample was set aside and 80% was used for training. Holdouts on each iteration were stratified such that there was a minimum of one of each responder type was represented in the holdout set. Once trained, the model was then used to make predictions on the 20% unseen data. Two shuffle sets were created for comparison, one by randomizing the labels in the training set prior to training, which was then used to make predictions on the same 20% unseen data, and one by randomizing the labels on the unseen data, which was then shown to the true labeled model to make predictions.

### Statistics and reproducibility

Statistical analyses were performed using GraphPad Prism (V10). Each distribution was assessed for normality with the D’Agostino-Pearson test, and the outcome was used to determine if parametric (for normal distributions) or non-parametric (non-normal) tests were most appropriate, as described below. In cases where multiple independent tests were performed on one set of data (e.g., three separate one-sample tests to compare three groups independently against a theoretical distribution), a non-parametric test was used for all comparisons if any one of the distributions did not pass the normality test or if the *n* of any one of the distributions were too small to test for normality (less than 8). For one-sample comparisons against a theoretical distribution, a one-sample Student’s *t* test was used in cases where assumptions of normality were met, and a Wilcoxon signed rank test was used in cases where assumptions were violated. For comparisons of a single dependent variable across three or more independent groups, a one-way ANOVA and Šídák’s multiple comparisons test were used when assumptions of normality were met, and a Kruskal–Wallis test and Dunn’s multiple comparisons test were used in cases where assumptions were violated. For comparisons of a single dependent variable sampled from the same subjects across three or more conditions, a one-way repeated measures ANOVA (or a mixed effects analysis when any values were missing) and Šídák’s multiple comparisons test were used when assumptions of normality were met, and a repeated measures Friedman’s test and Dunn’s multiple comparisons test were used in cases where assumptions were violated. In cases where data were sampled in a hierarchical/nested design, normality was assumed and a nested ANOVA was used to account for pseudoreplication. For comparisons with multiple categorical independent variables, an ordinary or repeated measures two-way ANOVA was used without testing for normality, as there are no equivalent non-parametric tests that satisfactorily test for interactions between independent variables (in all cases, subsequent subset analyses included normality testing as described above). Sphericity was never assumed, and a Greenhouse-Geisser correction was applied when tests of sphericity failed.

All tests were two-tailed and *p* values less than 0.05 were considered statistically significant. For all comparisons, main effects, and interactions, associated test statistic, *p* value, and degrees of freedom (where applicable) are detailed in the figure legends regardless of whether significant effects were detected.

## Results

### Opioid-targeted compounds modulate ethanol intake

**Fig. 1:**
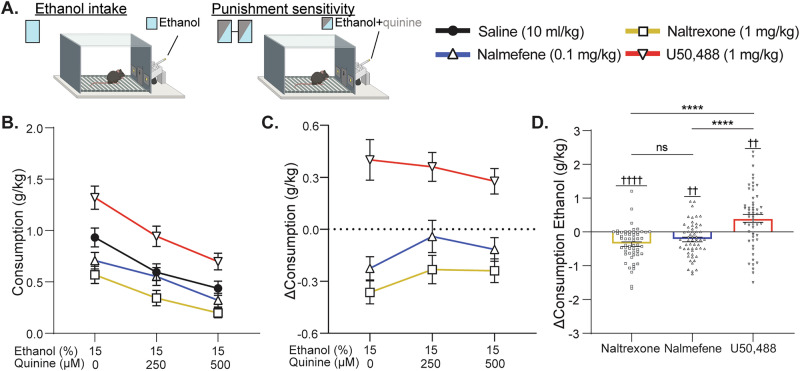
Opioid-targeted compounds bidirectionally modulate unpunished ethanol intake. **A** After acquisition of operant responding for ethanol, response to pharmacological compounds was assessed over the course of 3 sessions of ethanol self-administration on a fixed-ratio 10 schedule. The first session was unpunished responding for 15% ethanol, the next two sessions assessed punishment sensitivity with treatment with the addition of quinine (250 µM, 500 µM, consecutive sessions) to the ethanol solution. Each subject repeated this block for treatment with saline (vehicle), naltrexone (antagonist, 1.0 mg/kg), nalmefene (partial agonist, 0.1 mg/kg), and U50,488 (full agonist, 1.0 mg/kg). Data distributions are shown in Supplementary Figs. 4, 5. **B** Ethanol consumption over the three treatment sessions for each of the four treatments (*N* = 56). Consumption varied as a function of both treatment and quinine concentration, with no interaction between the two variables (repeated measures mixed-effects model; treatment: *F*_(3, 219)_ = 15.81, *p* < 0.0001; quinine concentration: *F*_(1.865, 406.5)_ = 58.79, *p* < 0.0001; treatment x quinine: *F*_(6, 436)_ = 1.15, *p* = 0.33). **C** Within-subject change in consumption from saline was calculated for each treatment across each of the three self-administration sessions. Change in consumption varied by treatment but was not modulated by quinine concentration and there was no interaction between treatment and concentration (repeated measures mixed-effects model; treatment: *F*_(1.747, 96.07)_ = 49.76, *p* < 0.0001; quinine concentration: *F*_(1.779, 97.87)_ = 0.65, *p* = 0.51; treatment × quinine: *F*_(2.644, 142.1)_ = 1.61, *p* = 0.20). Data distributions are shown panel **D** and Supplementary Figs. 4, 5. **D** The change in behavior was then compared to zero change to determine whether compounds modulated ethanol intake during unpunished sessions. During unpunished ethanol self-administration, naltrexone and nalmefene decreased consumption, whereas U50,488 increased intake compared to the vehicle control sessions (naltrexone, one-sample *t*-test, H_0_ = 0, *t*_55_ = 5.49, *p* < 0.0001; nalmefene, one-sample *t*-test, H_0_ = 0, *t*_55_ = 3.38, *p* = 0.0013; U50,488, one-sample *t*-test, H_0_ = 0, *t*_54_ = 3.21, *p* = 0.0012). U50,488 increased consumption more than naltrexone and nalmefene, but there was no difference in the reduction of consumption between naltrexone and nalmefene (Mixed-effects analysis, *F*_(1.6, 87)_ = 31.79, *p* < 0.0001; Šídák’s multiple comparisons: naltrexone vs nalmefene, *t*_55_ = 1.94, *p* = 0.16; naltrexone vs U50,488, *t*_54_ = 6.69, *p* < 0.0001; nalmefene vs U50,488, *t*_54_ = 5.40, *p* < 0.0001). Values indicate mean ± SEM. Statistical tests were two-sided. *N* = 56 mice; *p < 0.05; **p < 0.01; ***p < 0.001; ****p < 0.0001; *t*-test to zero: ^†^*p* < 0.05; ^††^*p* < 0.01; ^†††^*p* < 0.001; ^††††^*p* < 0.0001 vs. 0.

Both naltrexone and nalmefene are antagonists at mu and delta opioid receptors, but are thought to differ with respect to action at kappa opioid receptors; naltrexone typically acts as a neutral kappa opioid receptor antagonist (i.e., 0% intrinsic efficacy) while nalmefene acts as a partial agonist with roughly 25% efficacy compared to a full agonist^25^, though values can vary by preparation. To probe whether any observed differences between nalmefene and naltrexone might be due to differential intrinsic efficacy at kappa opioid receptors, a selective kappa opioid receptor full agonist, U50,488, was included in the study design. Following acquisition of operant responding (Supplementary Figs. 1, 2), male C57BL/6J mice were allowed to self-administer in daily one-hour sessions under a fixed-ratio 10 schedule reinforced by extension of a sipper containing alcohol for 10 s. Initial sessions were performed without treatment interventions to first establish baseline levels of alcohol intake in sessions wherein the sipper contained 15% ethanol (v/v), and punishment sensitivity wherein the alcohol was adulterated with the bitter tastant quinine. During the treatment phase, subjects were tested using a within-subject pseudorandomized block design with four blocks: placebo (0.9% saline, 10 mL/kg), naltrexone (1 mg/kg), nalmefene (0.1 mg/kg), and U50,488 (1 mg/kg). All subjects were tested under all four treatment block conditions. Order assignments were made by cage (5 subjects per cage) using a random number generator, such that each treatment appears at least once in each block order position. Treatments were delivered via intraperitoneal injection 30 min prior to self-administration sessions, and subjects were tested in 3 ethanol self-administration sessions per treatment block – one unadulterated ethanol session, one low-dose quinine session (250 µM), and one moderate-dose quinine session (500 µM) (Fig. 1A).

The effect of each treatment on consummatory behaviors was assessed and as expected, the compounds induce changes in ethanol intake (g/kg) that are modulated by quinine concentration (Fig. 1B). The within-subject change in unpunished ethanol consumption (Δ g/kg) was then calculated for each drug from each subject’s levels of intake when treated with saline. Aggregate analyses across all subjects revealed main effects of treatment compound and quinine concentration on ethanol consumption, but no interaction was detected, suggesting that these compounds modulate punished and unpunished ethanol intake to a similar degree (Fig. 1C). Naltrexone and nalmefene treatment reduced, whereas U50,488 increased, consumption during the unpunished session (Fig. 1D). In a critical comparison, there was no difference in the ability of naltrexone versus nalmefene to reduce ethanol consumption (Fig. 1D).

We were also able to assess the effect of these compounds on a variety of other AUD-relevant behaviors including: responses on the active operandum, a measure of appetitive behavior; and responses on the inactive operandum, a measure of generalized effects on non-ethanol reinforced behaviors (Supplementary Fig. 3). U50,488 increased the number of active responses made during self-administration sessions reinforced by ethanol alone, suggesting that kappa opioid receptor agonism can increase appetitive behaviors for ethanol (Supplementary Fig. 3C). None of the compounds altered responding on the inactive lever, thus it is unlikely that modulation of ethanol consumption occurs via generalized effects on activity (Supplementary Fig. 3D). No differences between nalmefene and naltrexone were detected for any of the outcome measures across punished and unpunished sessions with the exception of responding on the active and inactive operandum during the 500 µM and 250 µM quinine sessions, respectively (Supplementary Figs. 4, 5). Interestingly, the change in AUD-relevant behaviors was proportional to the estimated intrinsic efficacy of each compound at kappa opioid receptors (Supplementary Fig. 6). In sum, when assessed in all subjects in aggregate, opioid-targeted compounds bidirectionally modulate ethanol self-administration behaviors similarly across unpunished and punished contexts.

### Nalmefene and naltrexone are efficacious in distinct subpopulations

**Fig. 2:**
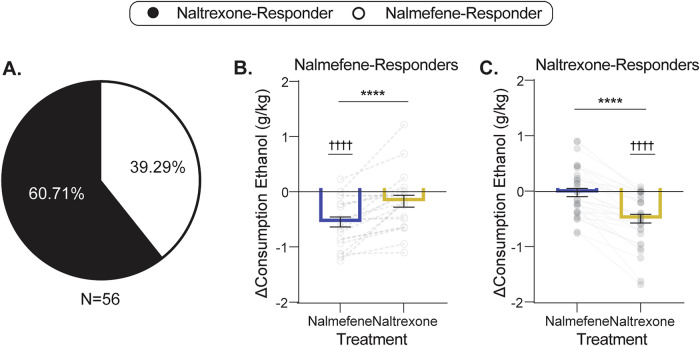
Subpopulations distinguished by responsiveness to nalmefene versus naltrexone display no therapeutic response to the alternative compound. **A** The proportion of subjects that showed a greater response to nalmefene or naltrexone: 60.71% responded more to naltrexone, and 39.29% responded more to nalmefene. **B** Change in consumption with naltrexone and nalmefene treatment for the nalmefene-responder subgroup. Nalmefene reduced consumption to a greater degree than naltrexone in nalmefene-responders (paired *t*-test, *t*_21_ = 4.80, *p* < 0.0001). Nalmefene induced a decrease in intake in nalmefene-responders (nalmefene, one-sample *t*-test, H_0_ = 0, *t*_21_ = 5.98, *p* < 0.0001), but there was no effect of naltrexone treatment on consumption in this subgroup (naltrexone, one-sample *t*-test, H_0_ = 0, *t*_21_ = 1.63, *p* = _0_.12). **C** Change in consumption with naltrexone and nalmefene treatment for the naltrexone-responder subgroup. Naltrexone reduced consumption to a greater degree than nalmefene in naltrexone-responders (paired *t*-test, *t*_33_ = 8.31, *p* < 0.0001). Naltrexone induced a decrease in intake in naltrexone-responders (one-sample *t*-test, H_0_ = 0, *t*_33_ = 6.19, *p* < 0.0001), but there was no effect of nalmefene treatment on consumption in this subgroup (one-sample *t*-test, H_0_ = 0, *t*_33_ = 0.25, *p* = 0.81). Values indicate mean ± SEM. Statistical tests were two-sided. naltrexone-responders, *n* = 34; nalmefene-responders, *n* = 22; *p < 0.05; **p < 0.01; ***p < 0.001; ****p < 0.0001; *t*-test to zero: ^†^*p* < 0.05; ^††^*p* < 0.01; ^†††^*p* < 0.001; ^††††^*p* < 0.0001 vs. 0.

The analyses above indicate that both nalmefene and naltrexone reduce drinking, but are essentially indistinguishable regarding effect size and actions on drinking-related behaviors at the population level. We next sought to determine if responsivity to these compounds is also indistinguishable at the individual level. At the individual level, once the decision to initiate a treatment is made, the optimal choice between two compounds can be considered as a binary: whether nalmefene or naltrexone is more effective in reducing ethanol drinking behaviors for a given subject. Accordingly, we did not designate a minimum delta required to be considered a responder; rather, each subject was deemed to be a naltrexone-responder or a nalmefene-responder based on which drug resulted in the lowest ethanol intake irrespective of magnitude. If both compounds resulted in zero consumption (9% of subjects), licks and responses on the active operandum were used to determine the preferred treatment. Of 56 subjects, 34 (60.7%) were naltrexone-responders and 22 (39.3%) were nalmefene-responders (Fig. 2A).

If nalmefene and naltrexone are in fact indistinguishable, we would expect to see similar effects regardless of subgroup, as the most treatment responsive subjects would have a large response to both compounds. In contrast, dividing the aggregate data reveals that, in nalmefene-responders, not only is the response to nalmefene greater than the response to naltrexone, but there is actually no effect of naltrexone on alcohol consumption within the subgroup (Fig. 2B). Likewise, in naltrexone-responders, naltrexone reduces drinking and nalmefene has no effect (Fig. 2C). Nalmefene and naltrexone-responders do not differ in response to U50,488 (Supplementary Fig. 7). Together, these results show that not only are nalmefene and naltrexone distinguishable at the individual level, but their efficacy in toto appears to be driven by orthogonal subpopulations which are unresponsive to the non-preferred treatment.

**Fig. 3:**
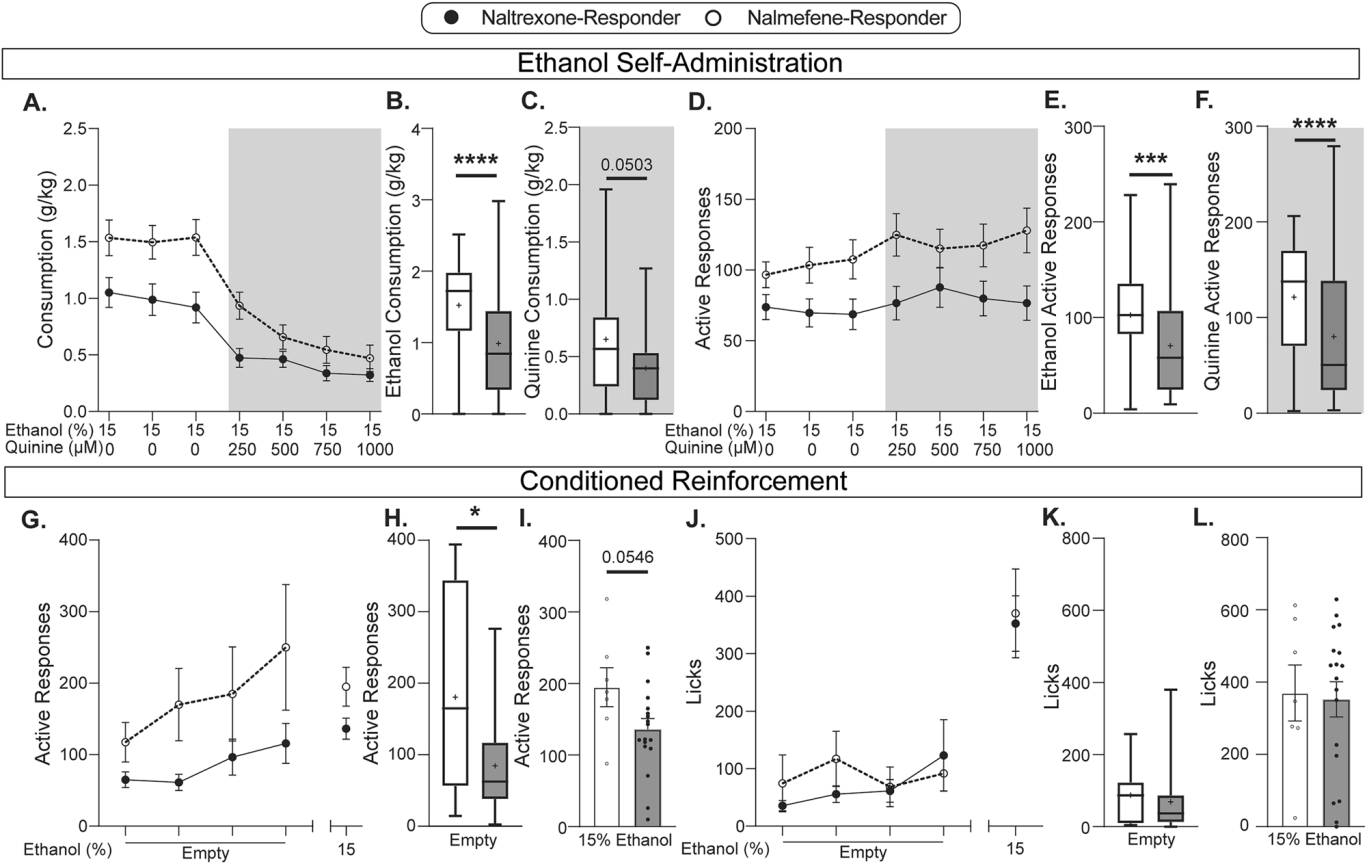
Subpopulations distinguished by responsiveness to nalmefene versus naltrexone display differential behavioral profiles across AUD-relevant domains. **A** Ethanol consumption across the 7 baseline sessions split by treatment response profile. Data distributions are shown in panels (**B**) and (**C**). **B**, **C** Box and whiskers plot showing inter-quartile ranges of nested consumption with the mean indicated by a cross. **B** Ethanol consumption over unpunished sessions varied by the most effective treatment with nalmefene-responders drinking more than naltrexone-responders during unpunished sessions (nested *t*-test, *t*_165_  =  4.48, *p* < 0.0001; 2 groups, 3 days per group, 167 total values). **C** Ethanol intake during punished phenotyping sessions showed a trending effect in the same direction, with nalmefene-responders consuming more ethanol adulterated with quinine than naltrexone-responders (nested *t*-test, *t*_6_  =  2.44, *p* = 0.05; 2 groups, 4 days per group, 224 total values). **D** Responses on the active operanda across phenotyping sessions, separated by treatment response profile. Data distributions are shown in panels (**E**) and (**F**). **E**, **F** Box and whiskers plot showing inter-quartile ranges of nested active responses with the mean indicated by a cross. **E** Nalmefene-responders made more active responses during ethanol-only sessions than naltrexone-responders (nested *t*-test, *t*_166_  =  3.54, *p* = 0.0005; 2 groups, 3 days per group, 168 total values). **F** In sessions where ethanol intake was punished with quinine, nalmefene-responders made more active responses than naltrexone-responders (nested *t*-test, *t*_222_  =  4.21, *p* < 0.0001; 2 groups, 4 days per group, 224 total values). **G**–**L** A subset of subjects underwent 4 conditioned reinforcement sessions during which the sipper was completely empty, followed by a single ethanol reintroduction session where responding was once again reinforced by 15% ethanol. **G** Active responses across the 4 conditioned reinforcement and ethanol reintroduction sessions separated by optimal treatment group. Data distributions are shown in panels (**H**) and (**I**). **H** Box and whiskers plot showing inter-quartile ranges of nested active responses with the mean indicated by a cross. Nalmefene-responders made more active responses during conditioned reinforcement sessions than naltrexone-responders (nested *t*-test, *t*_6_  =  3.59, *p* = 0.012; 2 groups, 4 days per group, 100 total values). **I** Active responding during the ethanol reintroduction session showed a trending effect in the same direction, with nalmefene-responders making more active responses than naltrexone-responders (unpaired *t*-test, *t*_23_ = 2.03, *p* = 0.055). **J** Number of licks across the 4 conditioned reinforcement sessions and the ethanol reintroduction session separated by most effective treatment. Data distributions are shown in panels (**K**) and (**L**). **K** Box and whiskers plot showing inter-quartile ranges of nested licks with the mean indicated by a cross. There was no difference in number of licks during conditioned reinforcement between nalmefene- and naltrexone-responders (nested *t*-test, *t*_6_  =  0.62, *p* = 0.56; 2 groups, 4 days per group, 100 total values). **L** There was no difference in the number of licks during the ethanol reintroduction session between nalmefene- and naltrexone-responders (unpaired *t*-test, *t*_23_ = 0.19, *p* = 0.85). Values indicate mean ± SEM, unless otherwise noted. Statistical tests were two-sided. naltrexone-responders, *n* = 34; nalmefene-responders, *n* = 22; *p < 0.05; **p < 0.01; ***p < 0.001; ****p < 0.0001.

To determine whether these subpopulations were meaningfully distinct, we then assessed whether there were any differences in consummatory and appetitive behavior during ethanol self-administration, prior to treatment (Fig. 3A–L). Following acquisition of operant responding but before treatment, mice underwent 7 days of baseline testing: 3 days assessing ethanol intake, and 4 days assessing punishment sensitivity during which the 15% (v/v) ethanol solution was adulterated with increasing concentrations of the bitter tastant quinine (250–1000 µM). We found that nalmefene-responders consumed more ethanol (Fig. 3A–C) and made more active responses (Fig. 3D–F) than naltrexone-responders during baseline sessions. To determine whether the two subpopulations differ across AUD-relevant behavioral domains, subjects were tested during conditioned reinforcement and reinstatement, thought to model processes related to seeking and relapse. To this end, a subset of subjects was run on 4 conditioned reinforcement sessions during which responding for an empty sipper tube was recorded. Subjects then underwent a reinstatement session where the sipper was once again filled with 15% ethanol. Nalmefene-responders make more active responses than naltrexone-responders during conditioned reinforcement sessions (Fig. 3G–I). Interestingly, despite the difference in responding for ethanol-conditioned stimuli, there was no difference between nalmefene- and naltrexone-responders in the number of licks during conditioned reinforcement (made on the dry spout) or reinstatement (when re-access to ethanol was given) (Fig. 3K, L). Thus, subpopulations with different treatment responsivity are characterized by divergent drinking and seeking behaviors, prior to treatment intervention. Ultimately, the distinct behavior profiles between nalmefene- and naltrexone-responders lend credence to the notion that these two compounds, despite similar mechanisms of action, are efficacious in two distinct, identifiable subpopulations.

### Preexisting drinking phenotype predicts most effective treatment

**Fig. 4:**
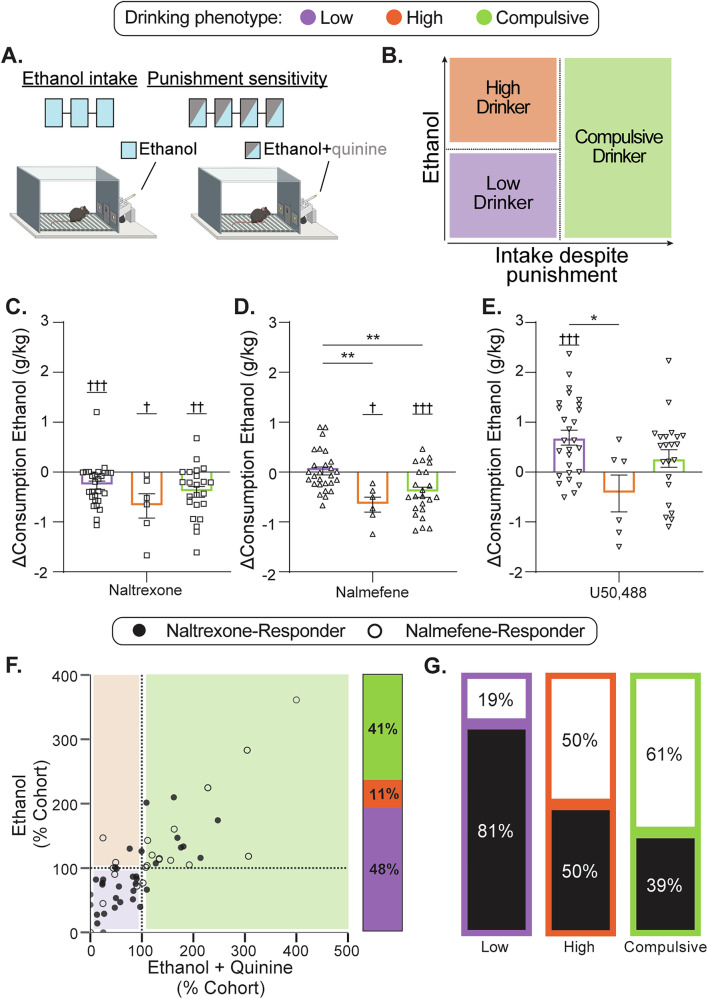
Nalmefene and naltrexone responders are differentially represented across drinking phenotypes. **A** After acquiring an ethanol reinforcement task subjects underwent 7 consecutive days of STAR phenotyping under a fixed-ratio 10 schedule for 10 s of sipper access. The first three sessions assessed ethanol intake, where the sipper contained 15% ethanol. Then, subjects underwent 4 sessions in which the sipper was filled with a 15% ethanol solution that was adulterated with an increasing dose of the bitter tastant, quinine (250, 500, 750, 1000 µM), to assess punishment sensitivity. **B** The average ethanol consumption across the three unpunished sessions and the average ethanol consumption across the four punished sessions were calculated for each subject and normalized to the cohort average. Subjects that drank more than average despite punishment were deemed Compulsive Drinkers, those that drank below average during punished sessions but above average during unpunished sessions were High Drinkers, and those that drank below average during both punished and unpunished sessions were Low Drinkers. **C**–**E** Change in consumption caused by each compound, separated by STAR phenotype. **C** Naltrexone decreased ethanol consumption in Low Drinkers (one-sample Wilcoxon test, H_0_ = 0, W = −255, *p* = 0.0002, *n* = 27), High Drinkers (one-sample Wilcoxon test, H_0_ = 0, W = −21, *p* = 0.03, *n* = 6), and Compulsive Drinkers (one-sample Wilcoxon test, H_0_ = 0, W = −175, *p* = 0.001, *n* = 23). There was no difference in effect of naltrexone between STAR phenotypes (Kruskal–Wallis test, H_2_ = 2.68, *p* = 0.26). **D** Nalmefene did not change consumption in Low Drinkers (one-sample Wilcoxon test, H_0_ = 0, W = −21, *p* = 0.8, *n* = 27), and decreased consumption in both High (one-sample Wilcoxon test, H_0_ = 0, W = −21, *p* = 0.03, *n* = 6) and Compulsive Drinkers (one-sample Wilcoxon test, H_0_ = 0, W = −191, *p* = 0.0004, *n* = 23). Nalmefene showed phenotype differences in efficacy (Kruskal–Wallis test, H_2_ = 14.15, *p* = 0.0008) with a greater reduction in consumption in High and Compulsive Drinkers than Low Drinkers and no difference between High and Compulsive Drinkers (Dunn’s multiple comparisons: low vs high, Z_(27, 6)_ = 3.05, *p* = 0.007; low vs compulsive, Z_(27, 23)_ = 2.99, *p* = 0.008; high vs compulsive, Z_(6, 23)_ = 1.15, *p* = 0.75). **E** U50,488 only increased consumption in Low Drinkers (one-sample Wilcoxon test, H_0_ = 0, W = 273, *p* = 0.0002, *n* = 27), not in High (one-sample Wilcoxon test, H_0_ = 0, W = −9, *p* = 0.44, *n* = 6) or Compulsive Drinkers (one-sample Wilcoxon test, H_0_ = 0, W = 73, *p* = 0.25, *n* = 22). U50,488 differentially affected phenotypes (Kruskal–Wallis test, H_2_ = 6.61, *p* = 0.04) with U50,488 increasing consumption in Low Drinkers more than High Drinkers (Dunn’s multiple comparisons, Z_(27, 6)_ = 2.48, *p* = 0.04), with no difference compared to Compulsive Drinkers (Dunn’s multiple comparisons, Z_(27, 22)_ = 1.35, *p* = 0.53) or between High and Compulsive drinkers (Dunn’s multiple comparisons: low vs high, Z_(6, 22)_ = 1.59, *p* = 0.34). **F** The normalized distributions of ethanol intake (y-axis) and ethanol intake during quinine sessions (x-axis) from baseline self-administration sessions with the group average intake indicated with a dotted line. The percent distribution of each phenotype. Of the 56 subjects, 41% were Compulsive Drinkers (*n* = 23), 11% were High Drinkers (*n* = 6), and 48% were Low Drinkers (*n* = 27). **G** The proportion of naltrexone- versus nalmefene-responders in each phenotype subgroup. The likelihood of being a nalmefene-responder is different for each phenotype and greatest in Compulsive Drinkers (chi-square, X^*2*^ = 9.66, *p* = 0.008). Values indicate mean ± SEM, unless otherwise noted (**D**, **E**, **G**, **H**). Statistical tests were two-sided. *N* = 56; Compulsive Drinkers, *n* = 23; High Drinkers, *n* = 6; Low Drinkers, *n* = 27. *p < 0.05; **p < 0.01; ***p < 0.001; ****p < 0.0001; *t*-test to zero: ^†^*p* < 0.05; ^††^*p* < 0.01; ^†††^*p* < 0.001; ^††††^*p* < 0.0001 vs. 0.

Each of the interventions had clear effects on ethanol intake in aggregate, however, reflecting what has been observed in clinical populations, there is wide variability in responses to each of the treatments across individuals. Given that this variability appears to be driven by divergent subpopulations across compounds, we next asked whether preexisting drinking phenotypes meaningfully map onto differential responsiveness to the pharmacological interventions. To this end, we implemented the Structured Tracking of Alcohol Reinforcement (STAR) framework, a robust and flexible model that allows for quantitative assessment of various AUD-relevant behavioral domains emphasized in the RDoC initiative. From the 7 baseline sessions, subjects were classified into three groups based on their average unpunished (ethanol only) and average punished (ethanol and quinine) intake (g/kg) (Fig. 4A). Drinkers with below average unpunished and punished intake are considered “Low Drinkers” (*n* = 27), whereas subjects with above average unpunished and below average punished consumption are deemed “High Drinkers” (*n* = 6). Those with above-average unpunished and punished consumption are categorized as “Compulsive Drinkers” (*n* = 23) indicating both heightened consumption and punishment insensitivity (Fig. 4B). STAR phenotyping stratifies the wide individual differences into clearly distinct subgroups across a range of measurements (Supplementary Fig. 8A–F). Phenotype assignments were made based on testing prior to treatment and were applied as a static designation throughout the dataset. Importantly, explicit a priori evaluation of hypotheses generated from this phenotyping framework in mice have found a striking degree of translation to human drinkers^57–61^.

Naltrexone decreased ethanol consumption across all phenotypes to a similar degree (Fig. 4C). Nalmefene, on the other hand, decreased consumption in High and Compulsive Drinkers but had no effect in Low Drinkers (Fig. 4D). U50,488 increased consumption only in Low Drinkers, with no effect in High and Compulsive Drinkers (Fig. 4E). Similar to the aggregate analysis, within each phenotype drug-induced changes in consumption are similar across unpunished and punished self-administration sessions (Supplementary Figs. 9, 10). Further, we found an orderly relationship between phenotype and treatment responsivity whereby the majority of Low Drinkers were naltrexone-responders, High Drinkers were evenly split between naltrexone- and nalmefene-responders, and the majority of Compulsive Drinkers were nalmefene-responders (Fig. 4F, G). These results mirror clinical findings, which suggest that nalmefene is most beneficial in patients with the highest drinking levels, and further suggest that those with lower pre-treatment drinking levels may be preferentially responsive to naltrexone.

### Circulating biomarkers for treatment responsivity

**Fig. 5:**
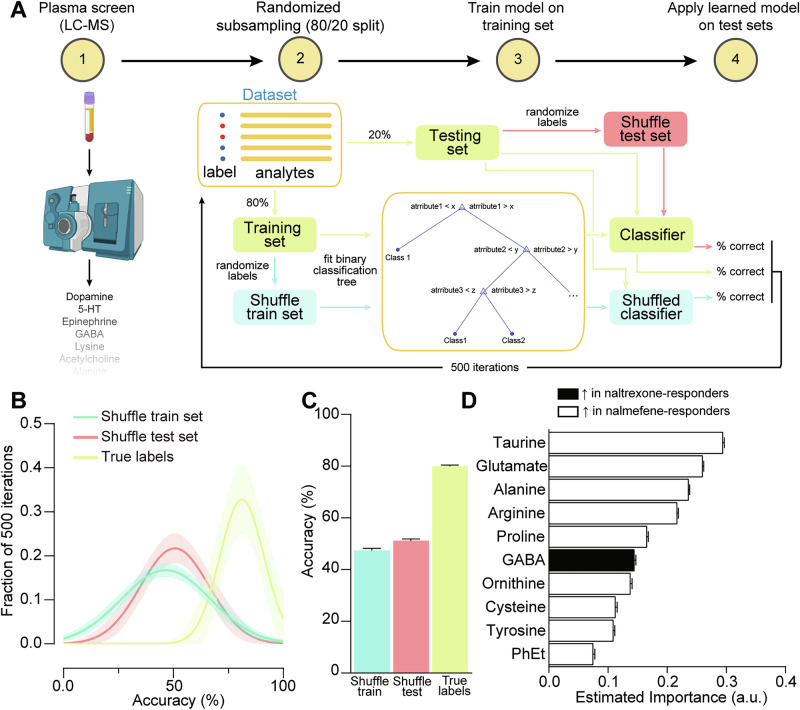
Optimal treatment compound can be predicted through circulating biomarker levels. **A** Schematic of predictive model pipeline. 1) A subset of subjects (*n* = 47) underwent three unpunished ethanol self-administration sessions. 24 h later, blood was collected and plasma was separated and 27 analytes were quantified via LC-MS. 2) The dataset was annotated with each animals’ classification as a nalmefene- or naltrexone-responder. A training set (80%) and test set (20%) were then created by randomly splitting the full dataset, and two shuffle sets were created by randomizing the labels in the training set or in the testing set. 3) A binary classification tree model was trained on the training set data only. A second model with identical input parameters was trained on the shuffled training data. 4) The true labeled model was then used to generate predictions for the test set (True label group) and shuffled test set data (Shuffle test set group). The model, which was trained on shuffled data, was used to generate predictions for the true labeled test set (Shuffle train set group). Predictions were then compared to the labels to determine the percent accuracy for each set. These values were stored and steps 2–4 were repeated for 500 iterations. **B** Fitted distributions of the percent accuracy over the 500 iterations for the shuffled training set, the shuffled test set, and the correctly labeled set. The shaded area represents the 95% confidence interval of the best-fit values. **C** Across iterations, the model achieved 80% accurate classifications in the labeled set, while only 47% and 51% were correctly classified in the shuffle training set and shuffled test set groups, respectively. **D** Estimated importance of each of the analytes was determined on each iteration and was then ranked based on the mean importance. The analytes with the top 10 highest scores are shown here. Filled vs open bars indicate whether analyte concentrations were higher in naltrexone- versus nalmefene-responders, respectively. GABA gamma-aminobutyric acid, PhEt. Unless otherwise indicated, values indicate mean ± SEM.

We next sought to determine whether treatment responsivity could be predicted via data obtained from a clinically-viable screening assay. We selected a blood-based screen as these samples are inexpensive and often are already collected in the clinic. 5–9 months after last pharmacological intervention, mice underwent three ethanol self-administration sessions, with the same parameters as an unpunished phenotyping session, and 24 h later blood was collected, and plasma analyzed via tandem liquid chromatography-mass spectrometry (LC-MS) for levels of circulating biogenic amines and amino acids (Fig. 5A). Concentrations of the 27 analytes measured are detailed in Supplementary Data 1. Based on the analyte concentration data, a binary classification decision tree model was trained to differentiate whether each subject was a naltrexone- or nalmefene-responder. The model was iteratively trained and tested using a 20% holdout permutation such that all testing was performed on unseen data. Two control conditions were also tested on each iteration, whereby a model was trained on data with shuffled labels and tested on the true label holdout and, separately, the true model was tested on the 20% holdout data but with shuffled labels to determine chance prediction accuracy (Fig. 5A). The mean accuracy across iterations was 47% for the shuffled training set condition and 51% for the shuffled testing set condition, demonstrating that neither control condition produces spurious predictions as they did not differ from chance. However, the model that was trained and tested on true labeled data was able to predict the most effective treatment with 80% accuracy (Fig. 5B, C). We also extracted the relative importance of each of the analytes for classification outcomes and found that taurine, glutamate, and alanine are the top three most important factors for classification and, for all three, higher concentrations of these biomarkers was associated with a nalmefene-responder classification (Fig. 5D). Together, these results provide multiple biomarkers which could readily transferred to clinical studies for assessment of forward translatability.

## Discussion

To date, there have been no clinical trials that directly compare the efficacy of naltrexone versus nalmefene, and there is ongoing debate as to the comparative efficacy of these two compounds as well as the regulatory status of nalmefene. Through a direct, within-subject comparison of the efficacy of naltrexone versus nalmefene for reducing ethanol consumption, we demonstrate that, on the population level, both naltrexone and nalmefene are similarly effective in reducing ethanol consumption. However, when the most effective compound is considered, we find that naltrexone is the most effective treatment for ~60% of subjects and nalmefene is the most effective treatment for ~40% of subjects. Importantly, these are two distinct subpopulations of individuals – nalmefene-responders show no response to naltrexone and vice versa. Our results suggest that, if prescribed using a personalized approach, utilizing both compounds may have an additive effect on overall treatment outcomes even if there is no clear difference in efficacy when each is assessed in isolation using a one-size-fits-all approach.

To provide a roadmap for how nalmefene and naltrexone could be optimized for improving AUD treatment, we took two approaches to predicting whether an individual would be more likely to be a naltrexone-responder or a nalmefene-responder—by phenotype classification and by individual biomarker levels. To determine whether phenotypic differences in drinking behaviors prior to treatment might predict responsivity, we utilize the STAR framework as it allows for intuitive stratification of individual differences into subgroups that have proven to be effective for animal-to-human translation^57–61^. We find that the likelihood of being a nalmefene-responder is highest in Compulsive Drinkers, and Low Drinkers are primarily naltrexone-responders. Finally, to predict the most effective treatment for a given individual, we utilize a blood-based screen whereby concentrations of 27 analytes are obtained from a single sample of plasma using liquid chromatography-mass spectrometry. We find that nalmefene- and naltrexone-responders can be distinguished with high accuracy based on levels of circulating analytes alone. While the blood-based screening approach would require the creation of a database of samples and clinical outcomes to train a predictive model, the behavioral predictors could potentially be implemented based on patient self-report. Critically, both methods provide a tractable starting point for attempting precision treatment strategies.

Both a benefit and limitation of this work is that the analytes (as they are assessed here) are correlative and predictive. We do not posit a mechanistic relationship between the blood analyte concentrations and treatment responsivity; and it is unlikely that manipulating the levels of these circulating analytes directly will change responsivity to treatment despite their predictive relationship in the endogenous state. Though we cannot make claims on the mechanism by which each analyte is associated with treatment response, this screening approach allows us to identify informative biomarkers without relying on preexisting data and hypotheses. Thus, this expedites clinical application as it would allow for predictive screening without relying on preclinical consensus on the mechanism by which the analyte is implicated in alcohol use. For example, though some of the analytes, like taurine, have been noted in the past as being associated with various aspects of drinking, work has produced conflicting results on whether the analyte increases or decreases alcohol consumption^62,63^. Here, we find that higher taurine blood levels are associated with being a nalmefene-responder but make no claim on if or how it causally contributes to the phenotype. This also provides a benefit in the field, such that it opens the door for mechanistic explorations of analytes not commonly associated with AUD. Notably, we only explored a small number of the potential analytes peripherally accessible – there is ongoing ‘omics’ work exploring how the metabolome, proteome, transcriptome, genome, and epigenome are associated with AUD risk, prognosis, and treatment response^9,64^.

Though structurally and pharmacodynamically similar, naltrexone and nalmefene are thought to differ in their therapeutic action via variable effects at the kappa opioid receptor. While polymorphisms in *OPRM1* (the gene encoding the mu opioid receptor) have been associated with differences in alcohol use disorder prevalence and naltrexone efficacy in reducing consumption and craving^7,65–70^, they do not appear to be associated with nalmefene response^71^, thus it is unlikely that it would be predictive of the most effective treatment for an individual. Instead, clinical and preclinical studies suggest that individual differences in treatment response for both compounds result from actions at the kappa opioid receptor, and in a direct comparison it was suggested that nalmefene is more effective specifically in ethanol-dependent subjects due to its activity at the kappa opioid receptor. Additionally, polymorphisms and differential gene expression of both the kappa opioid receptor and its endogenous ligand, dynorphin, have been associated with increased risk of AUD, and expression and function of kappa opioid receptors are upregulated by chronic ethanol exposure^55,72–75^. Together, these previous findings implicate differential action at kappa opioid receptors between nalmefene and naltrexone as a potential avenue for identifying advantageous approaches for precision application of these compounds, and to serve as a counterpoint for the pharmacodynamic profiles of nalmefene and naltrexone, we also administered the kappa opioid receptor agonist, U50,488.

Interestingly, the two drugs with agonistic properties at the kappa opioid receptor, nalmefene and U50,488, show diverging effects with nalmefene only reducing consumption in High and Compulsive Drinkers, and U50,488 only increasing consumption in Low Drinkers. Given that nalmefene is a partial agonist at kappa opioid receptors, depending on ongoing endogenous ligand binding it can result in agonism, if the endogenous agonist is low, or antagonism, if endogenous agonist is high and displaced by the partial agonist. Considering nalmefene produces similar effects to the antagonist naltrexone in High and Compulsive Drinkers, we speculate that these phenotypes have higher levels of endogenous kappa opioid receptor activity, presumably through dynorphin binding. In contrast, in Low Drinkers, there may be more moderate levels of endogenous signaling, minimizing the difference in kappa opioid receptor activity, and thus behavioral change, induced by the partial agonist. This framework would also predict that the largest effect of the full agonist would be observed in the subpopulation with the lowest baseline kappa opioid receptor activity, based on a larger delta in signaling induced by the drug. Thus, we hypothesize that dynorphin levels may differ between the phenotypes and play an important role in determining treatment responsivity. Another intriguing possibility is that differential activity at kappa opioid receptors is driven by disparate β-endorphin levels, which have been shown to differ between high and low drinking phenotypes^76–79^ and have low nanomolar affinity for kappa opioid receptors^25^.

Differential activity at kappa opioid receptors as an important determinant of AUD-relevant behaviors is congruent with previous literature suggesting that chronic ethanol intake induces upregulated kappa opioid receptor activity, which drives escalation of intake. Importantly, though, previous work has focused on kappa opioid receptor plasticity after induction of ethanol dependence through non-contingent ethanol vapor exposure, which occludes individual differences in drinking behaviors. Here, we highlight individual differences across subjects that have had access to ethanol under the same conditions but nevertheless develop different behavioral patterns during ethanol self-administration. It will be important in the future to directly measure endogenous dynorphin levels across timepoints to determine whether altered signaling is a preexisting risk factor for becoming a High or Compulsive Drinker, or if it is an effect of higher levels of ethanol intake and punishment insensitivity, in the case of the Compulsive Drinkers. In either case, here we provide preliminary evidence that differences in kappa opioid receptor signaling are associated with individual differences in AUD-relevant behavior and may contribute to treatment response. Importantly, these results in aggregate provide a framework for preclinical research to contribute to the mechanistic understanding of differential treatment responsivity.

### Limitations

It is critical to note the limitations associated with this study, as experiments were only conducted in male mice, a single strain, with one dose per compound, and with a short-term dosing regimen. Further, no single model can recapitulate the full spectrum of AUD-like behaviors. It will be crucial for future work to investigate individual differences in the response to nalmefene and naltrexone in females to inform the application of a precision medicine approach in this specific subpopulation. There is a wide range of literature demonstrating major sex differences in pharmacodynamics of kappa opioid receptor ligands, and the mechanisms of action of many compounds are now debated for female subjects^43–48^. We focused on male subjects given that the pharmacodynamics were characterized in males and there is general consensus as to the basic properties of kappa ligands in this population. Thus, the results outlined in this manuscript should not be generalized to females without further investigation. Though genetic polymorphisms, including those within opioid receptor genes, have been implicated in AUD rate, symptoms, and treatment response, we specifically chose to use the inbred C57BL/6 J strain to limit the genetic contribution to individual differences. Future work investigating the genetic contribution to treatment response could leverage this study design to probe different strains with more genetic variance. It is important to note that, despite the genetic homogeneity of the inbred strains, recent work has shown that C57BL/6J mice are as variable as outbred mice across a plethora of measures including behavioral outcomes and molecular signatures, suggesting that the individual variability observed within this study would be comparable to that of a genetically heterogenous population^80^. Additionally, nalmefene and naltrexone dosages were selected based on previous work demonstrating similar efficacy in reducing alcohol responding in rats^50^. Injections were also administered i.p., whereas in humans, both treatments are commonly administered orally. Both dosage and route of administration should be investigated in further translational investigations of this work.

## Conclusions

Precision medicine is rarely assessed in preclinical models, but this stage of testing is crucial as it allows for the investigations of novel therapeutics as well as provides the ability to assess the mechanistic role of specific systems in individual differences in disease presentation and treatment efficacy. In this work, we provided evidence that nalmefene may provide benefit among patients that are unresponsive to naltrexone despite their similar mechanisms of action. Further, nalmefene responders may potentially be identifiable through behavioral and plasma phenotypes. Together, this work provides insight into the mechanisms by which AUD-relevant behaviors are modulated, lays the groundwork for a preclinical precision AUD medicine pipeline, and reveals putative biomarkers for maximizing treatment efficacy.

## Supplementary information

Supplemental Figures

Supplementary Data 1

Supplementary Data 2

Description of Additional Supplementary Data

## Data Availability

Data types used in this manuscript include behavioral data and LC-MS analyte concentrations. LC-MS values are found in Supplementary Data 1. The source data for Figs. 1–4 can be found in Supplementary Data 2.
