# Supplementary material for: Nalmefene and naltrexone reduce alcohol intake via selective efficacy in subpopulations distinguished by behavioral and blood-based biomarkers
Source: Commun Med (Lond). 2026 Jan 14;6:106. doi: 10.1038/s43856-025-01369-6 (PMC12894949; doi:10.1038/s43856-025-01369-6)
Supplement: Supplementary file 1 — Supplemental Figures [file 43856_2025_1369_MOESM1_ESM.pdf]

Supplementary Materials for

Nalmefene and naltrexone reduce alcohol intake via selective efficacy in subpopulations distinguished by behavioral and blood-based biomarkers

Zahra Z. Farahbakhsh[†], Alex R. Brown[†], Suzanne O. Nolan, Snigdha Mukerjee, Cody A. Siciliano*

Vanderbilt University, Department of Pharmacology, Vanderbilt Brain Institute, Vanderbilt Center for Addiction Research, Nashville, TN 37232, USA.

[†]co-first authors

Corresponding author:
Cody A Siciliano
cody.siciliano@vanderbilt.edu

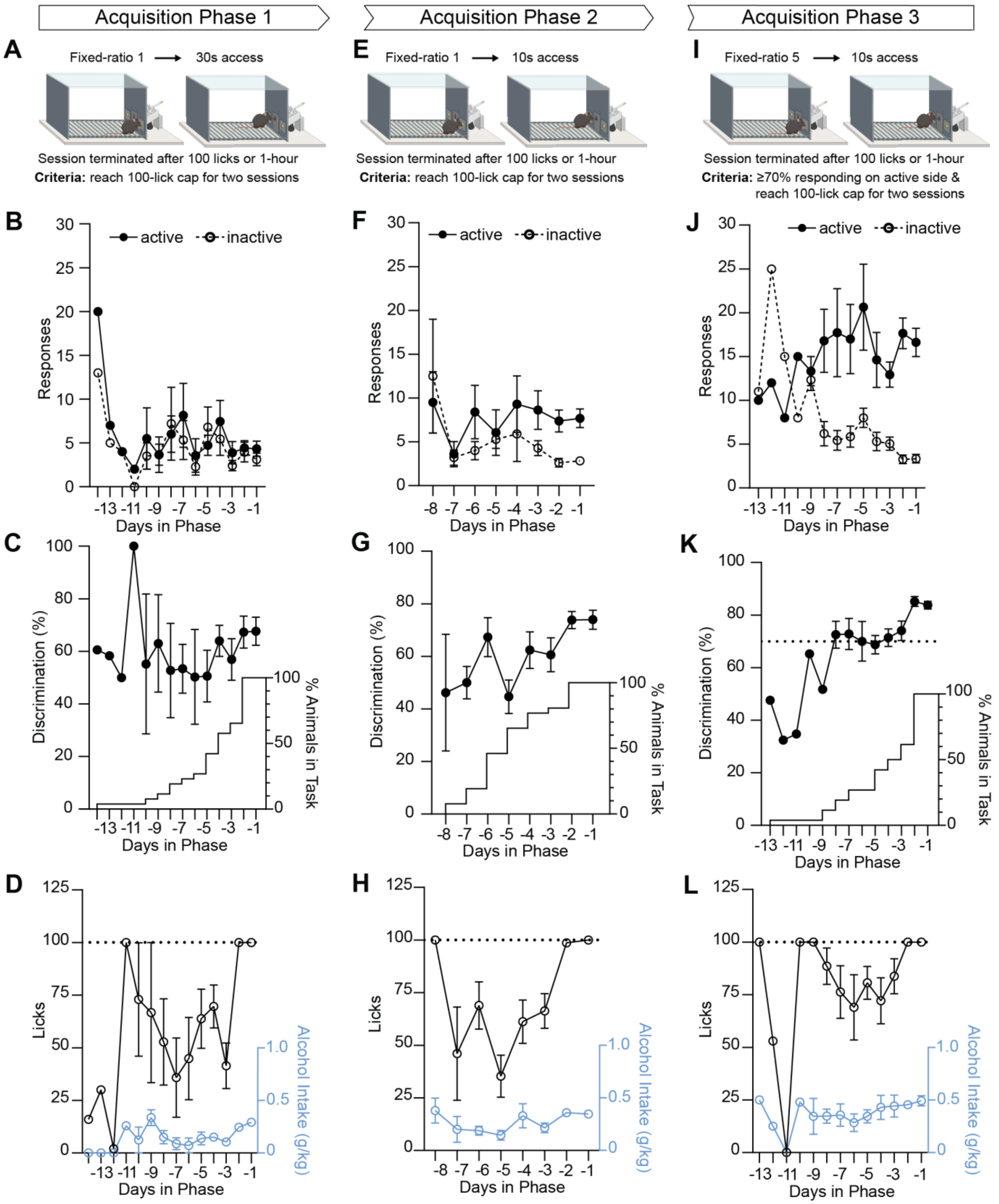

Supplementary Figure 1. Operant ethanol self-administration acquisition rates for cohort 1. (A) Acquisition Phase 1 experimental parameters and acquisition criteria. Responses were reinforced under a fixed-ratio 1 schedule by extension of the sipper for 30s. Sessions were terminated after 100 licks are reached on the lickometer or after one hour, whichever came first. **(B)** Active (solid line) and inactive (dotted line) operanda responses during Acquisition Phase 1. **(C)** Percent active operandum discrimination (active responses / [active + inactive responses]) during Acquisition Phase 1. Percentage of subjects remaining for each day is indicated on the right y-axis. **(D)** Licks and alcohol Consumption (g/kg) during Acquisition Phase 1.

100 lick acquisition criteria indicated by dotted line. **(E)** Acquisition Phase 2 experimental parameters and acquisition criteria. Responses were reinforced under a fixed-ratio 1 schedule by extension of the alcohol sipper for 10s. Sessions were terminated after 100 licks were reached on the lickometer or after one hour, whichever came first. **(F)** Total responses on either the active (solid line) or inactive (dotted line) operanda during Acquisition Phase 2. **(G)** Percent active operandum discrimination during Acquisition Phase 2. Percentage of subjects remaining for each day is indicated on the right y-axis. **(H)** Licks and alcohol Consumption (g/kg) during Acquisition Phase 2. 100 lick acquisition criteria indicated by dotted line. **(I)** Acquisition Phase 3 experimental parameters and acquisition criteria. Responses were reinforced under a fixed-ratio 5 schedule by extension of the alcohol sipper for 10s. Sessions were terminated after 100 licks were reached on the lickometer or after one hour, whichever came first. **(J)** Total responses on either the active (solid line) or inactive (dotted line) operanda during Acquisition Phase 3. **(K)** Percent active operandum discrimination during Acquisition Phase 3 – dotted line indicates response discrimination criteria in this phase. Percentage of subjects remaining for each day is indicated on the right y-axis. **(L)** Licks and alcohol intake (g/kg) during Acquisition Phase 3. 100 lick acquisition criteria indicated by dotted line. Values indicate mean ± SEM. n = 26, percent of the 26 subjects represented by each data point are indicated on the right y-axis of panels C, G, and K.

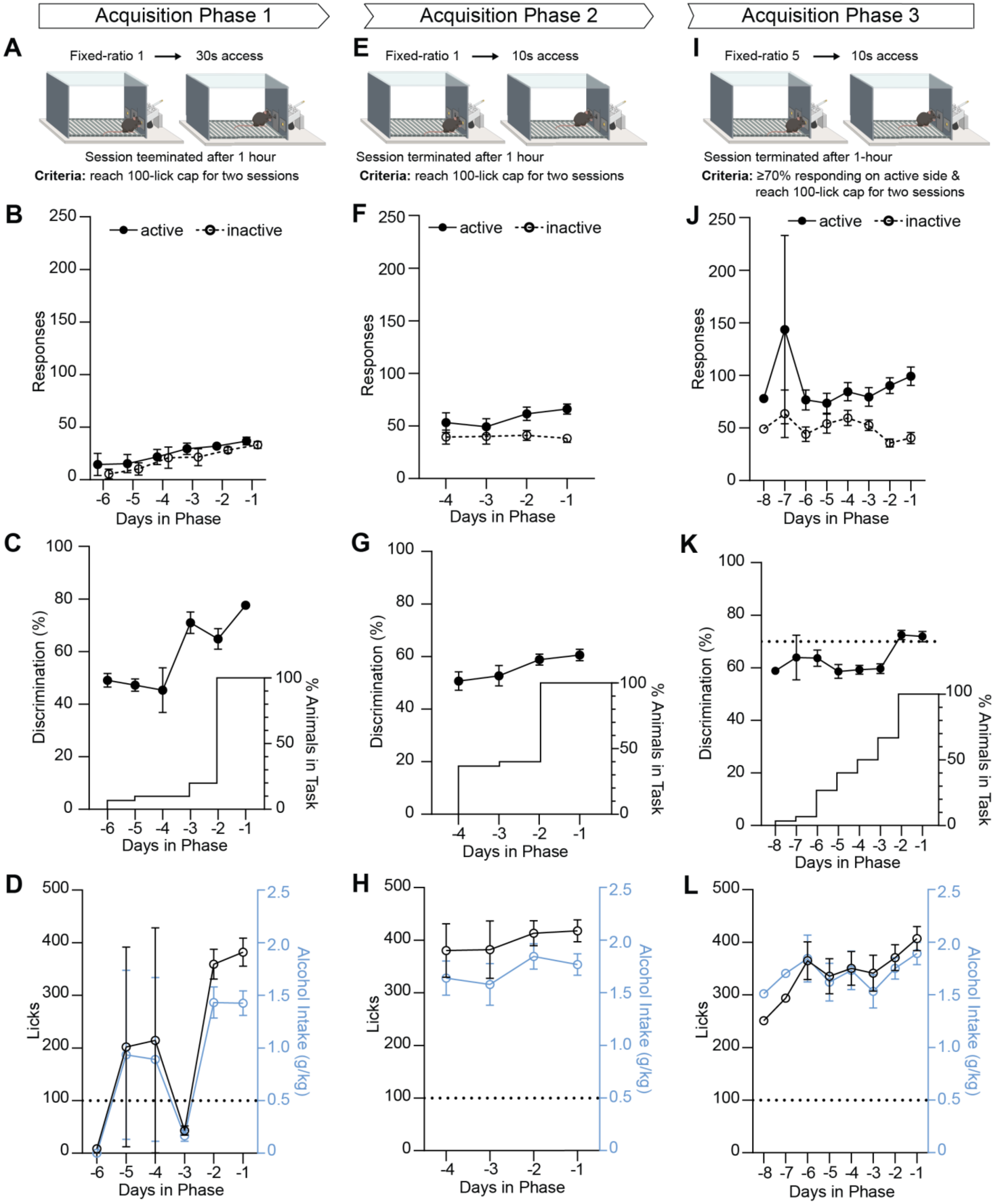

Supplementary Figure 2. Operant ethanol self-administration acquisition rates for cohort 2. (A) Acquisition Phase 1 experimental parameters and acquisition criteria for cohort 2. Responses were reinforced under a fixed-ratio 1 schedule by extension of the sipper for 30s. Sessions were terminated after one hour. **(B)** Active (solid line) and inactive (dotted line) responses during Acquisition Phase 1. **(C)** Percent active operandum discrimination (active responses / [active + inactive responses]) during Acquisition Phase 1.

Percentage of subjects remaining for each day is indicated on the right y-axis. **(D)** Licks and alcohol Consumption (g/kg) during Acquisition Phase 1. 100 lick acquisition criteria indicated by dotted line. **(E)** Acquisition Phase 2 experimental parameters and acquisition criteria. Responses were reinforced under a fixed-ratio 1 schedule by extension of the alcohol sipper for 10s. Sessions were terminated after one hour. **(F)** Total responses on either the active (solid line) or inactive (dotted line) operanda during Acquisition Phase 2. **(G)** Percent active operandum discrimination during Acquisition Phase 2. Percentage of subjects remaining for each day is indicated on the right y-axis. **(H)** Licks and alcohol Consumption (g/kg) during Acquisition Phase 2. 100 lick acquisition criteria indicated by dotted line. **(I)** Acquisition Phase 3 experimental parameters and acquisition criteria. Responses were reinforced under a fixed-ratio 5 schedule by extension of the alcohol sipper for 10s. Sessions were terminated after one hour. **(J)** Total responses on either the active (solid line) or inactive (dotted line) operanda during Acquisition Phase 3. **(K)** Percent active operandum discrimination during Acquisition Phase 3 – dotted line indicates response discrimination criteria in this phase. Percentage of subjects remaining for each day is indicated on the right y-axis. **(L)** Licks and alcohol intake (g/kg) during Acquisition Phase 3. 100 lick acquisition criteria indicated by dotted line. Values indicate mean ± SEM. n = 30, percent of the 30 subjects represented by each data point are indicated on the right y-axis of panels C, G, and K.

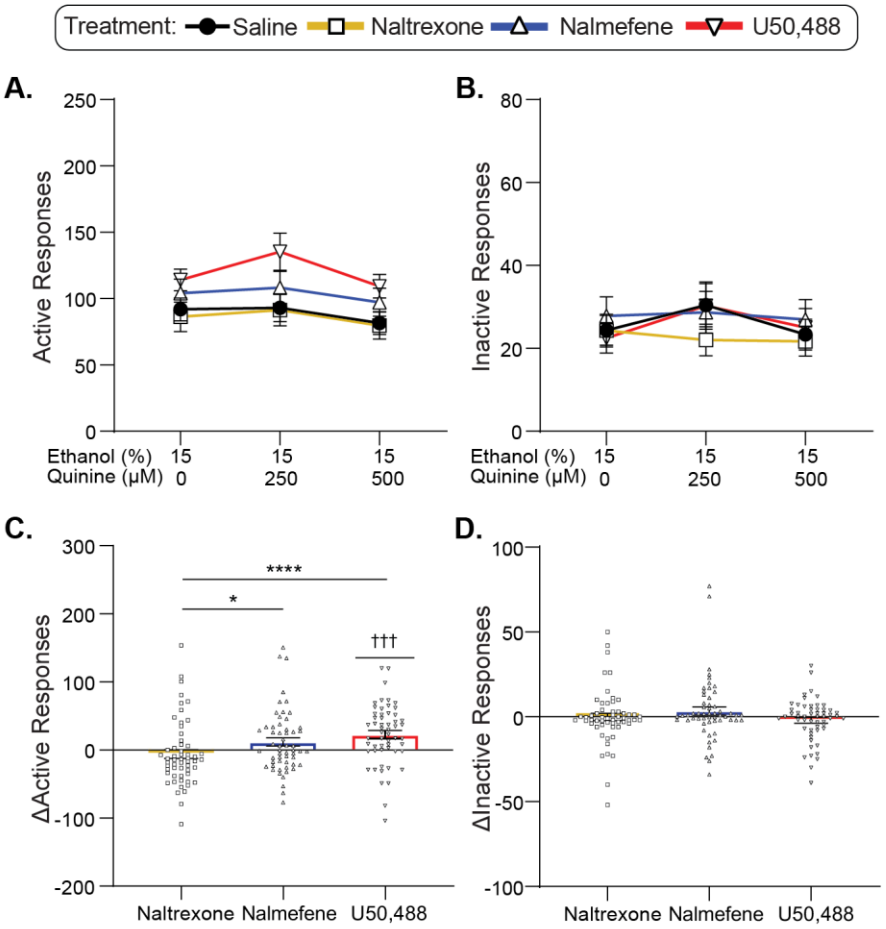

Supplementary Figure 3. Active and inactive responding across sessions and within-subject. (A) Active responses over the three treatment sessions for each of the four treatments. **(B)** Inactive responses during each treatment session across each of the four treatments. **(C)** During the unpunished sessions, naltrexone and nalmefene did not change the number of active responses from within-subject vehicle sessions, but U50,488 increased active responding (naltrexone, one-sample Wilcoxon test, $H_0 = 0$, $W = -442$, $p = 0.06$, $n = 56$; nalmefene, one-sample Wilcoxon test, $H_0 = 0$, $W = 345$, $p = 0.14$, $n = 56$; U50,488, one-sample Wilcoxon test, $H_0 = 0$, $W = 869$, $p = 0.0001$, $n = 55$). U50,488 and nalmefene increased active responding more than naltrexone (Friedman test, $R_2 = 22.82$, $p < 0.0001$; Dunn's multiple comparisons: naltrexone vs nalmefene, $Z_{(55, 55)} = 2.86$, $p = 0.01$; naltrexone vs U50,488, $Z_{(55, 55)} = 4.72$, $p < 0.0001$; nalmefene vs U50,488, $Z_{(55, 55)} = 1.86$, $p = 0.19$). **(D)** Naltrexone, nalmefene, and U50,488 administration did not alter the number of inactive responses during ethanol self-administration (naltrexone, one-sample Wilcoxon test, $H_0 = 0$, $W = -182$, $p = 0.42$, $n = 56$; nalmefene, one-sample Wilcoxon test, $H_0 = 0$, $W = 298$, $p = 0.23$, $n = 56$; U50,488, one-sample Wilcoxon test, $H_0 = 0$, $W = -144$, $p = 0.50$, $n = 55$). There was a difference in effect on inactive responding between treatments, but no two treatments were different from one another (Friedman test, $R_2 = 6.71$, $p = 0.03$; Dunn's multiple comparisons: naltrexone vs nalmefene, $Z_{(55, 55)} = 2.34$, $p = 0.06$; naltrexone vs U50,488, $Z_{(55, 55)} = 1.96$, $p = 0.15$; nalmefene vs U50,488, $Z_{(55, 55)} = 0.38$, $p > 0.99$). Values indicate mean ± SEM. Statistical tests were two-sided. N = 56 mice; *$p < 0.05$; **$p < 0.01$; ***$p < 0.001$; ****$p < 0.0001$; t-test to zero: $^{\dagger}p < 0.05$; $^{\dagger\dagger}p < 0.01$; $^{\dagger\dagger\dagger}p < 0.001$; $^{\dagger\dagger\dagger\dagger}p < 0.0001$ vs. 0.

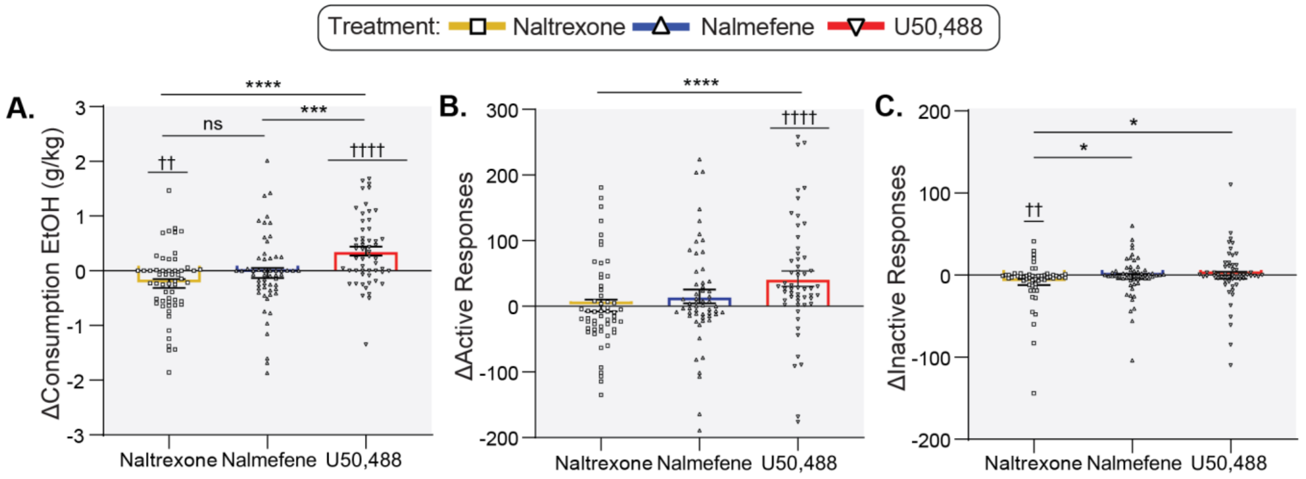

Supplementary Figure 4. Effect of treatment across behaviors during 250 µM quinine sessions. (A-C) Within subject change of behavioral measures during 250 µM treatment sessions compared to vehicle control. **(A)** Naltrexone led to an overall reduction of alcohol consumption, whereas nalmefene showed no change and U50,488 led to an overall increase in consumption (naltrexone, one-sample t-test, $H_0 = 0$, $t_{54} = 2.83$, p = 0.0066; nalmefene, one-sample t-test, $H_0 = 0$, $t_{55} = 0.45$, p = 0.66; U50,488, one-sample t-test, $H_0 = 0$, $t_{54} = 4.29$, $p < 0.0001$). There was no difference in change in punished consumption between nalmefene and naltrexone, but U50,488 increased consumption relative to both (Mixed-effects analysis, $F_{(2, 108)} = 16.96$, $p < 0.0001$; Šídák's multiple comparisons: naltrexone vs nalmefene, $t_{108} = 1.82$, p = 0.20; naltrexone vs U50,488, $t_{108} = 5.70$, $p < 0.0001$; nalmefene vs U50,488, $t_{108} = 3.91$, p = 0.0005). **(B)** Treatment with U50,488 showed an increase in active responses compared to vehicle, with no differences observed during treatment with naltrexone or nalmefene (naltrexone, one-sample Wilcoxon test, $H_0 = 0$, W = -182, p = 0.45, n = 56; nalmefene, one-sample Wilcoxon test, $H_0 = 0$, W = 290, p = 0.24, n = 56; U50,488, one-sample Wilcoxon test, $H_0 = 0$, W = 951, $p < 0.0001$, n =55). There was no difference in change in active responding between nalmefene and naltrexone, but U50,488 increased responding relative to naltrexone (Friedman test, $R_2 = 19.27$, $p < 0.0001$; Dunn's multiple comparisons: naltrexone vs nalmefene, $Z_{(54, 54)} = 2.26$, p = 0.07; naltrexone vs U50,488, $Z_{(54, 54)} = 4.38$, $p < 0.0001$; nalmefene vs U50,488, $Z_{(54, 54)} = 2.12$, p = 0.10). **(C)** Treatment with naltrexone decreased inactive responding relative to vehicle, with no difference observed with nalmefene or U50,488 treatment (naltrexone, one-sample Wilcoxon test, $H_0 = 0$, W = -704, p = 0.001, n = 55; nalmefene, one-sample Wilcoxon test, $H_0 = 0$, W = -53, p = 0.82, n = 56; U50,488, one-sample Wilcoxon test, $H_0 = 0$, W = 2, p = 0.99, n =55). . There was a difference in effect on inactive responding between the three treatments with naltrexone reducing active responding compared to both nalmefene and U50,488 (Friedman test, $R_2 = 10.33$, p = 0.006; Dunn's multiple comparisons: naltrexone vs nalmefene, $Z_{(54, 54)} = 2.45$, p = 0.04; naltrexone vs U50,488, $Z_{(54, 54)} = 2.74$, p = 0.02; nalmefene vs U50,488, $Z_{(54, 54)} = 0.29$, $p > 0.99$). Values indicate mean ± SEM. Statistical tests were two-sided. N = 56 mice; *p < 0.05; **p < 0.01; ***p < 0.001; ****p < 0.0001; t-test to zero: †p < 0.05; ††p < 0.01; †††p < 0.001; ††††p < 0.0001 vs. 0.

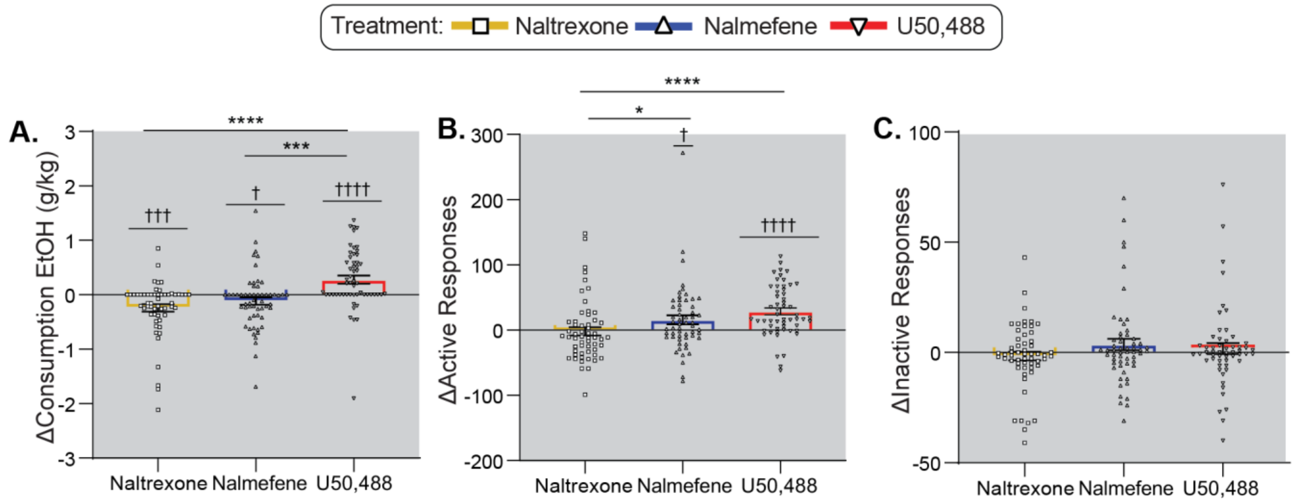

Supplementary Figure 5. Effect of treatment across behaviors during 500 μM quinine sessions. (A-C) Within subject change of behavioral measures during 500 μM treatment sessions compared to vehicle control. **(A)** Treatment with naltrexone decreased, whereas U50,488 increased, consumption compared to vehicle, while there were no changes observed with nalmefene treatment (naltrexone, one-sample Wilcoxon test, $H_0 = 0$, W = -538, p = 0.0002, n = 55; nalmefene, one-sample Wilcoxon test, $H_0 = 0$, W = -321, p = 0.04, n = 56; U50,488, one-sample Wilcoxon test, $H_0 = 0$, W = 664, p < 0.0001, n =55). There was no difference in change in punished consumption between nalmefene and naltrexone, but U50,488 increased consumption relative to both (Friedman test, $R_2 = 34.74$, p < 0.0001; Dunn's multiple comparisons: naltrexone vs nalmefene, $Z_{(54, 54)} = 1.01$, p = 0.94; naltrexone vs U50,488, $Z_{(54, 54)} = 4.91$, p < 0.0001; nalmefene vs U50,488, $Z_{(54, 54)} = 3.90$, p = 0.0003). **(B)** Active responses were increased after treatment with nalmefene and U50,488 relative to vehicle control, with no differences observed after naltrexone treatment (naltrexone, one-sample Wilcoxon test, $H_0 = 0$, W = -343, p = 0.16, n = 56; nalmefene, one-sample Wilcoxon test, $H_0 = 0$, W = 524, p = 0.03, n = 56; U50,488, one-sample Wilcoxon test, $H_0 = 0$, W = 1133, p < 0.0001, n =55). U50,488 and nalmefene increased active responding relative to naltrexone (Friedman test, $R_2 = 20.95$, p < 0.0001; Dunn's multiple comparisons: naltrexone vs nalmefene, $Z_{(55, 55)} = 2.05$, p = 0.12; naltrexone vs U50,488, $Z_{(55, 55)} = 4.53$, p < 0.0001; nalmefene vs U50,488, $Z_{(55, 55)} = 2.48$, p = 0.04). **(C)** No changes were observed in inactive responses after any of the treatments (naltrexone, one-sample Wilcoxon test, $H_0 = 0$, W = -162, p = 0.49, n = 56; nalmefene, one-sample Wilcoxon test, $H_0 = 0$, W = 174, p = 0.47, n = 56; U50,488, one-sample Wilcoxon test, $H_0 = 0$, W = 28, p = 0.90, n =55) and there was a difference in effect on inactive responding between treatments, but no two treatments were different from one another (Friedman test, $R_2 = 6.29$, p = 0.04; Dunn's multiple comparisons: naltrexone vs nalmefene, $Z_{(55, 55)} = 2.28$, p = 0.07; naltrexone vs U50,488, $Z_{(55, 55)} = 1.86$, p = 0.19; nalmefene vs U50,488, $Z_{(55, 55)} = 0.43$, p > 0.99). Values indicate mean ± SEM. Statistical tests were two-sided. N = 56 mice; *p < 0.05; **p < 0.01; ***p < 0.001; ****p < 0.0001; t-test to zero: †p < 0.05; ††p < 0.01; †††p < 0.001; ††††p < 0.0001 vs. 0.

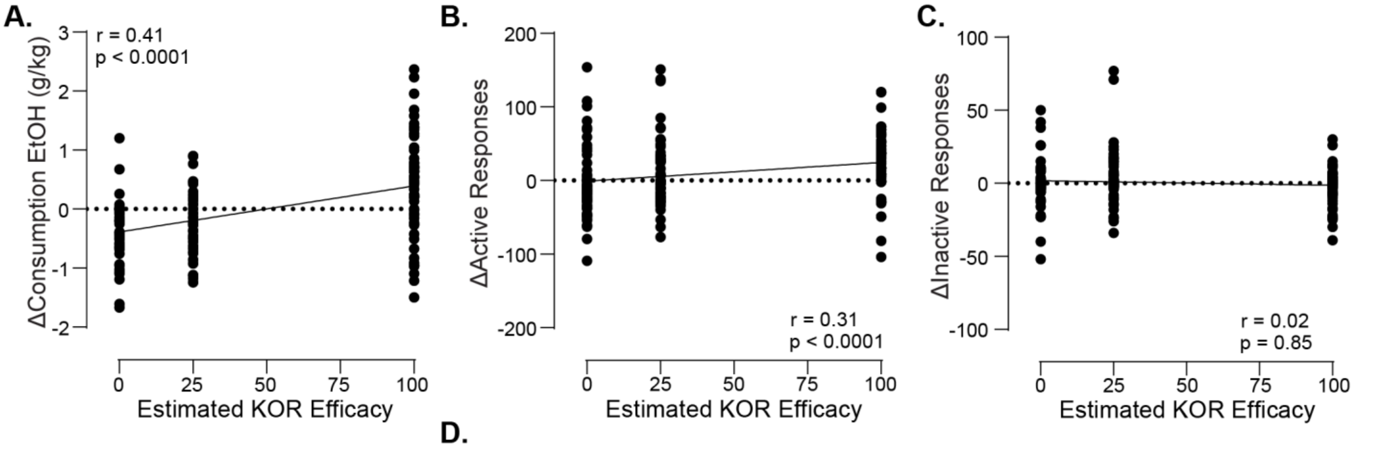

Supplementary Figure 6. Modulation of ethanol reinforced behaviors tracks approximated KOR pharmacodynamics. To test the relationship between pharmacodynamic profile at KORs and modulation of drinking behavior, we correlated the estimated KOR efficacy for naltrexone (0% efficacy), nalmefene (25% efficacy), and U50,488 (100% efficacy) with their effect on each of the outcome measures. **(A-C)** Change in behavior from pharmacological intervention (y-axis) by putative efficacy from the literature at the KOR of each compound (x-axis). Best fit linear curve is shown. The r and p-value from spearman's correlation indicated in inset. **(A)** There was a correlation between the change in consumption and estimated KOR efficacy. **(B)** The change in active responding correlated with estimated KOR efficacy. **(C)** There was no relationship between inactive responding and estimated efficacy at the KOR. N = 56 mice.

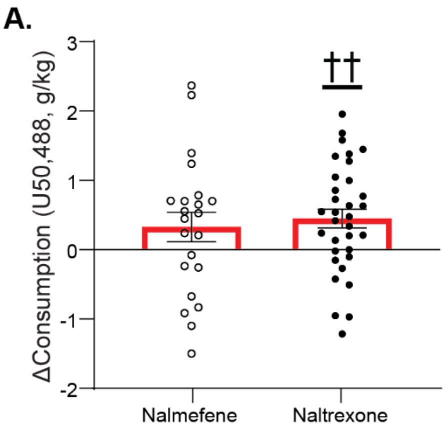

Supplementary Figure 7. Effect of U50,488 on consumption in nalmefene versus naltrexone responders. (A) While there was no difference in change in consumption with U50,488 treatment in nalmefene versus naltrexone responders (unpaired t-test, $t_{53} = 0.51$, $p = 0.61$), U50,488 only increased consumption in naltrexone-responders (one-sample t-test, $H_0 = 0$, $t_{32} = 3.28$, $p = 0.0025$), not nalmefene-responders (one-sample t-test, $H_0 = 0$, $t_{21} = 1.54$, $p = 0.14$). Values indicate mean ± SEM. Statistical tests were two-sided naltrexone-responders, n = 33; nalmefene-responders, n = 22; $^{††}p < 0.01$.

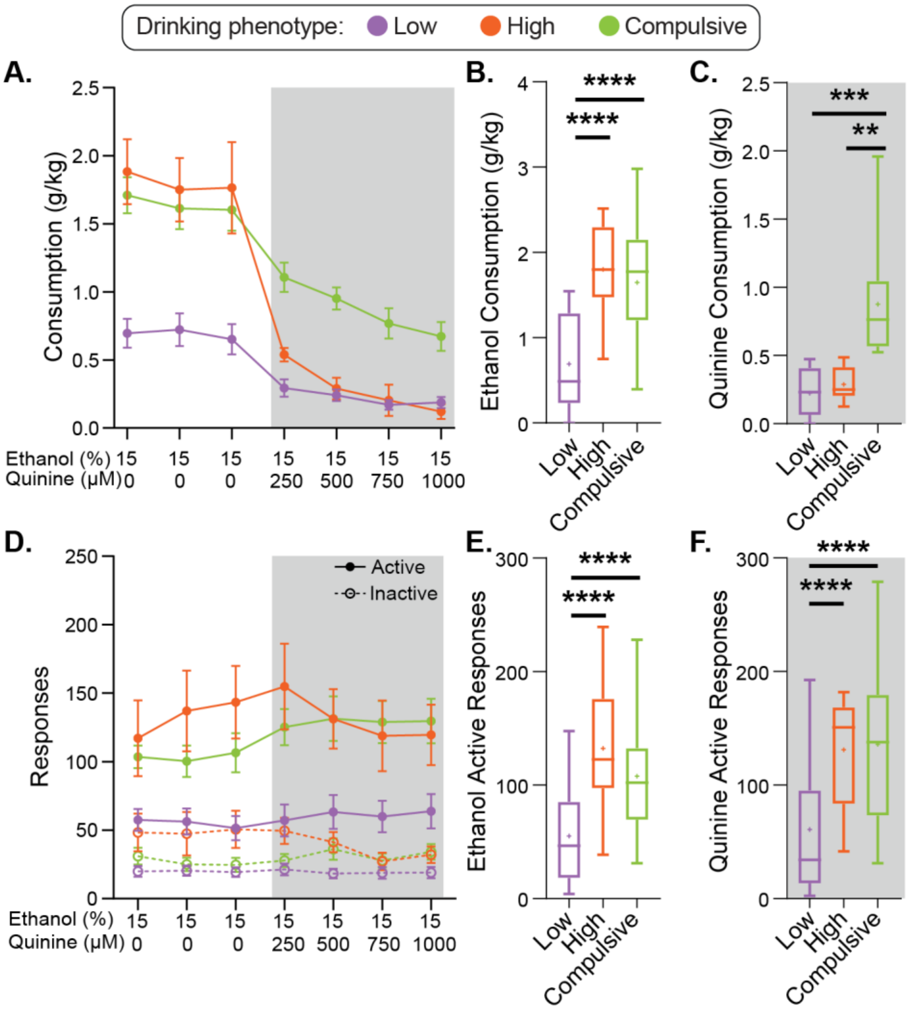

Supplementary Figure 8. STAR phenotyping separates three behaviorally distinct populations. (A) Ethanol consumption across the 7 phenotyping sessions split by phenotype. **(B-C)** Box and whiskers plot showing inter-quartile ranges of nested consumption with the mean indicated by a cross. **(B)** Ethanol consumption over unpunished sessions varied by phenotype (nested one-way ANOVA, $F_{(2, 164)} = 51.90$, $p < 0.0001$; 3 groups, 3 days per group, 167 total values) with no difference between High and Compulsive Drinkers (Tukey's test, High vs. Compulsive: $p = 0.67$), but both groups consuming more than Low Drinkers (Tukey's test, High vs. Low: $p < 0.0001$; Compulsive vs. Low: $p < 0.0001$). **(C)** Consumption of ethanol adulterated with increasing concentrations of quinine was different between phenotypes (nested one-way ANOVA, $F_{(2, 9)} = 25.08$, $p = 0.0002$; 3 groups, 4 days per group, 224 total values). Compulsive Drinkers consumed more than High and Low Drinkers (Tukey's test, Compulsive vs. Low: $p = 0.0002$; Compulsive vs. High: $p = 0.002$), with no difference in consumption between Low and High Drinkers (Tukey's test, High vs. Low: $p = 0.84$). **(D)** Responses on the inactive and active operanda across phenotyping sessions, separated by phenotype. **(E-F)** Box and whiskers plot showing inter-quartile ranges of nested active responses with the mean indicated by a cross. **(E)** Active responses during unpunished ethanol sessions was different between phenotypes (nested one-way ANOVA, $F_{(2, 165)} = 29.44$, $p < 0.0001$; 3 groups, 3 days per group, 168 total values) as High and Compulsive Drinkers made more active responses than Low Drinkers (Tukey's test, High vs. Low: $p < 0.0001$; Compulsive vs. Low: $p < 0.0001$), but did not differ from each other (Tukey's test, High vs. Compulsive: $p = 0.16$). **(F)** Active responses during quinine adulterated sessions differed by phenotype (nested one-way ANOVA, $F_{(2, 221)} = 35.37$, $p < 0.0001$; 3 groups, 4 days per group, 224 total values). Again, Compulsive and High phenotypes did not differ (Tukey's test, High vs. Compulsive: $p = 0.94$), but both groups responded more than Low Drinkers (Tukey's test, High vs. Low: $p < 0.0001$; Compulsive vs. Low: $q_{221} = 11.35$, $p < 0.0001$). Values indicate mean ± SEM, unless otherwise noted (B, C, E, F). Statistical tests were two-sided. N

= 56; Compulsive Drinkers, n = 23; High Drinkers, n = 6; Low Drinkers, n = 27. N = 56; *p < 0.05; **p < 0.01; ***p < 0.001; ****p < 0.0001.

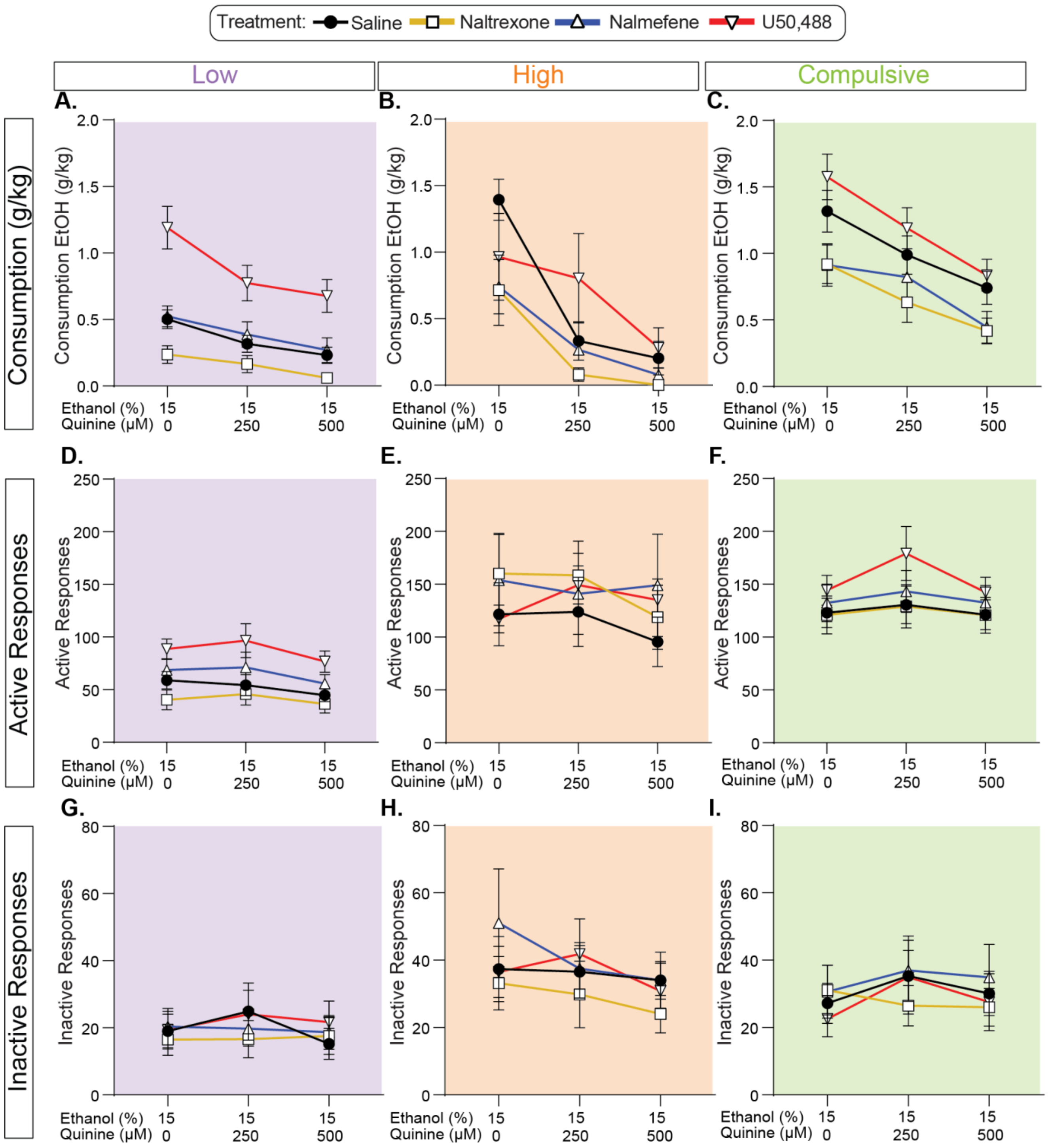

Supplementary Figure 9. Behavioral measures across treatments and quinine concentration separated by phenotype. (A-C) Consumption of ethanol (g/kg) under different treatment conditions and quinine concentrations for Low **(A)**, High **(B)**, and Compulsive **(C)** drinking phenotypes. Active **(D-F)** and Inactive **(G-I)** responses for ethanol solutions under different treatment conditions for Low **(D & G)**, High **(E & H)**, and Compulsive **(F & I)** drinking phenotypes. Values indicate mean ± SEM. N = 56; Compulsive Drinkers, n = 23; High Drinkers, n = 6; Low Drinkers, n = 27.

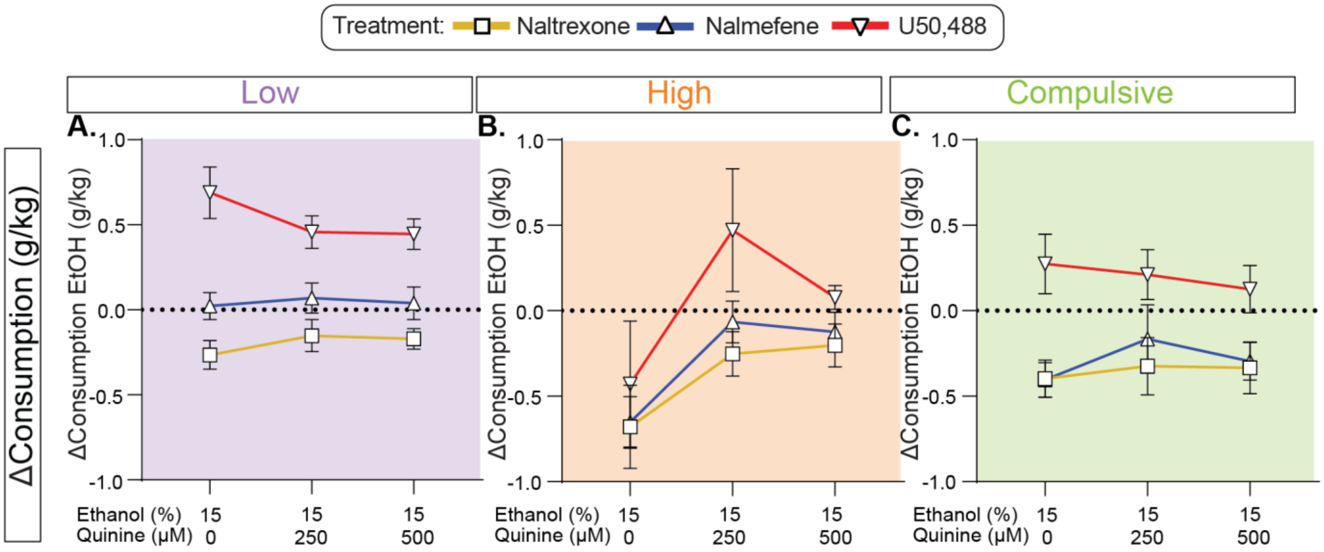

Supplementary Figure 10. There is no effect of quinine concentration or interaction between quinine and treatment on the change in consumption for any phenotype. (A-C) Change in ethanol consumption over the three treatment sessions for each treatment relative to the vehicle treatment split by phenotype. **(A)** In Low Drinkers, change in consumption varied as a function of treatment, with no effect of quinine concentration or interaction between the two variables (two-way ANOVA; treatment: $F_{(1.435, 37.31)} = 30.51$, $p < 0.0001$; quinine: $F_{(1.829, 47.56)} = 0.15$, $p = 0.84$; treatment x quinine: $F_{(2.392, 62.18)} = 1.99$, $p = 0.14$). **(B)** In High Drinkers, there were no effects observed of treatment, quinine, or interaction between the two variables (two-way ANOVA; treatment: $F_{(1.468, 7.341)} = 3.81$, $p = 0.081$; quinine: $F_{(1.272, 6.360)} = 5.11$, $p = 0.057$; treatment x quinine: $F_{(1.439, 7.193)} = 0.42$, $p = 0.61$). **(C)** Compulsive Drinkers displayed an effect of treatment on consumption, with no effect of quinine or interaction between the two variables. (mixed-effects analysis; treatment: $F_{(1.981, 43.58)} = 16.27$, $p < 0.0001$; quinine: $F_{(1.736, 38.19)} = 0.18$, $p = 0.80$; treatment x quinine: $F_{(2.999, 62.22)} = 0.66$, $p = 0.58$). Values indicate mean ± SEM. Statistical tests were two-sided. Low Drinkers n = 27; High Drinkers n = 6; Compulsive Drinkers n = 23.
