# Supplementary material for: Nalmefene and naltrexone reduce alcohol intake via selective efficacy in subpopulations distinguished by behavioral and blood-based biomarkers
Source: Commun Med (Lond). 2026 Jan 14;6:106. doi: 10.1038/s43856-025-01369-6 (PMC12894949; doi:10.1038/s43856-025-01369-6)
Supplement: Supplementary file 4 — Description of Additional Supplementary Data [file 43856_2025_1369_MOESM4_ESM.docx]

Description of additional supplementary file

File name: Supplementary Data 1

Description: LC-MS values are found in Supplementary Data 1.

File name: Supplementary Data 2

Description: The source data for figures 1-4 can be found in Supplementary Data 2.
